# Identification of ROCK1 as a novel biomarker for postmenopausal osteoporosis and pan-cancer analysis

**DOI:** 10.18632/aging.205004

**Published:** 2023-09-07

**Authors:** Bowen Lai, Heng Jiang, Yuan Gao, Xuhui Zhou

**Affiliations:** 1 Department of Orthopedics, Changzheng Hospital, Second Military Medical University, Shanghai, China

**Keywords:** postmenopausal osteoporosis, ROCK1, machined Learning, immune infiltration, pan-cancer

## Abstract

Background: Postmenopausal osteoporosis (PMOP) is a prevalent bone disorder with significant global impact. The elevated risk of osteoporotic fracture in elderly women poses a substantial burden on individuals and society. Unfortunately, the current lack of dependable diagnostic markers and precise therapeutic targets for PMOP remains a major challenge.

Methods: PMOP-related datasets GSE7429, GSE56814, GSE56815, and GSE147287, were downloaded from the GEO database. The DEGs were identified by “limma” packages. WGCNA and Machine Learning were used to choose key module genes highly related to PMOP. GSEA, DO, GO, and KEGG enrichment analysis was performed on all DEGs and the selected key hub genes. The PPI network was constructed through the GeneMANIA database. ROC curves and AUC values validated the diagnostic values of the hub genes in both training and validation datasets. xCell immune infiltration and single-cell analysis identified the hub genes’ function on immune reaction in PMOP. Pan-cancer analysis revealed the role of the hub genes in cancers.

Results: A total of 1278 DEGs were identified between PMOP patients and the healthy controls. The purple module and cyan module were selected as the key modules and 112 common genes were selected after combining the DEGs and module genes. Five Machine Learning algorithms screened three hub genes (KCNJ2, HIPK1, and ROCK1), and a PPI network was constructed for the hub genes. ROC curves validate the diagnostic values of ROCK1 in both the training (AUC = 0.73) and validation datasets of PMOP (AUC = 0.81). GSEA was performed for the low-ROCK1 patients, and the top enriched field included protein binding and immune reaction. DCs and NKT cells were highly expressed in PMOP. Pan-cancer analysis showed a correlation between low ROCK1 expression and SKCM as well as renal tumors (KIRP, KICH, and KIRC).

Conclusions: ROCK1 was significantly associated with the pathogenesis and immune infiltration of PMOP, and influenced cancer development, progression, and prognosis, which provided a potential therapy target for PMOP and tumors. However, further laboratory and clinical evidence is required before the clinical application of ROCK1 as a therapeutic target.

## INTRODUCTION

Osteoporosis is a significant global health concern characterized by low bone mineral density (BMD), heightened bone fragility, and an increased risk of fracture [1]. Postmenopausal osteoporosis (PMOP), in particular, imposes a considerable physical and financial burden on aging women, with an estimated one-third of women over fifty experiencing fractures induced by osteoporosis [2]. Additionally, osteoporosis-related fractures, predominantly in the hip, spine, and wrist, resulting in more hospitalization days for women over 45 than other chronic diseases, including diabetes, heart disease, and breast cancer [2]. After a hip fracture, the quality of life significantly declines, with 40% of patients unable to walk independently, and 20-24% of patients passing away within the first year [2]. Consequently, early diagnosis and treatment of osteoporosis are critical, particularly for postmenopausal women who face a high risk of developing the condition.

Currently, the most widely used diagnostic method for osteoporosis is the bone mineral density (BMD) test, which employs dual-energy X-ray absorptiometry (DXA). Other diagnostic techniques include radiological assessments and Bone Turnover Markers (BTM) [3]. However, due to the absence of systemic symptoms until the occurrence of a fracture, these approaches may not detect early or atypical cases of PMOP. Recently, the advent of genome-wide association studies (GWAS) and multi-omics techniques has enabled the identification of a variety of genes, such as RANKL and ESR1, associated with PMOP susceptibility. This discovery has expanded early diagnostic strategies for the disease, including specific gene testing [4, 5].

The treatment for PMOP should be selected based on the severity of bone mass loss and the risk of fracture. For low-risk women, non-pharmacological interventions such as calcium and vitamin D supplementation, regular exercise, and abstaining from smoking and drinking are recommended [6, 7]. For patients requiring drug therapy, anti-resorptive agents, including bisphosphonates, raloxifene, and denosumab, can decrease bone destruction, while anabolic agents, such as teriparatide, promote bone formation [1, 8, 9]. Nevertheless, these drugs have significant adverse effects that can pose considerable risks to patients’ health. As such, the optimal therapeutic options for PMOP are still under further investigation [10]. Therefore, there is an urgent need to explore the hallmark genes that are closely associated with the onset and progression of PMOP to enhance the precision of therapy for this condition.

The conventional theory regarding the pathogenesis of PMOP suggests that it is primarily caused by aging and estrogen deficiency, which results in a phenotype of increased bone loss and decreased bone remodeling [11]. Nonetheless, the immune system’s role in the development of PMOP has garnered significant attention, leading to the introduction of a novel term, immunoporosis, to examine the intricate relationship between the skeletal and immune systems in osteoporosis pathogenesis [12]. Research has suggested that aging or low estrogen levels result in the continuous activation of the immune system at low levels, causing immune balance disturbance, promoting osteoclast activation, and ultimately leading to bone loss [13, 14]. Conducting a comprehensive analysis of immune cell activity and function in PMOP, particularly changes in immune-related molecular markers, can contribute to the expansion of the scope of PMOP etiology research, thus advancing the application of immunotherapy for the disease. Cancer is a severe disease with high morbidity and mortality [15]. The possible shared etiology of aging, disturbed hormone levels, and immune infiltration has prompted an exploration of the relationship between cancers and PMOP. Recent studies have reported that PMOP patients have a higher incidence of cancer, such as breast cancer, while impaired bone health and a heightened risk of fracture are frequently observed in advanced cancer, resulting in a poor prognosis [16–18]. However, few studies have investigated the shared molecular mechanisms of the two diseases. Further investigation into potential PMOP biomarkers, putative pathogenesis, and shared treatment targets between PMOP and cancer is thus warranted.

Bioinformatics has emerged as a promising cross-disciplinary tool for investigating pathophysiological mechanisms of diseases. One such tool is Weighted Gene Co-expression Network Analysis (WGCNA), which can identify gene modules with similar expression patterns and screen out modules containing key regulatory genes that are highly correlated with disease phenotypes [19]. Furthermore, Machine Learning algorithms, which possess powerful computing and sorting capabilities, can be utilized to select disease-related genes [20]. Another bioinformatics technique, xCell, can evaluate immune cell composition in diseases [21]. While some studies have identified hub genes associated with primary osteoporosis (PMOP) [22, 23], few have combined WGCNA and Machine Learning to select hub genes and focus on immune infiltration levels and related biomarkers.

**Figure 1:**
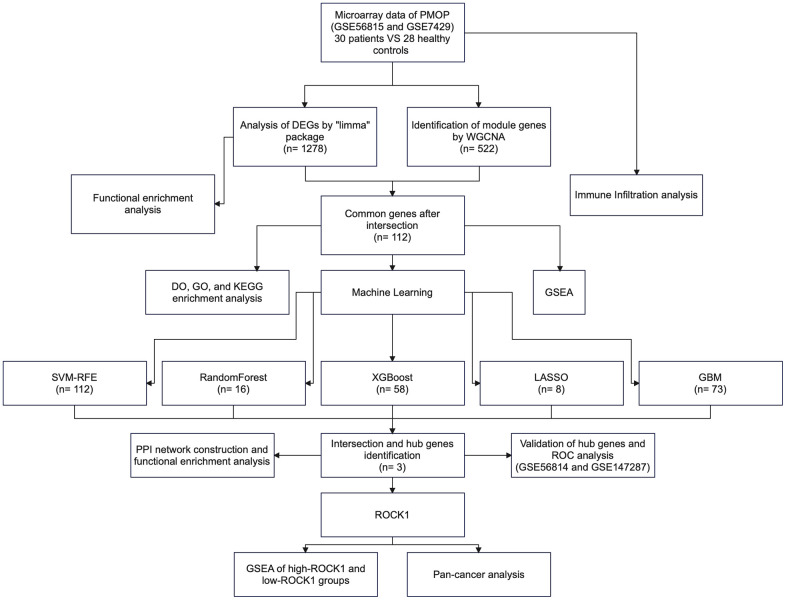
The flowchart of the study process.

In this study (Figure 1), we utilized the Gene Expression Omnibus (GEO) database to download the mRNA expression datasets GSE56815 and GSE7429 from PMOP patients and identified differential expression genes (DEGs). By employing the cross-disciplinary tools of WGCNA and Machine Learning, we further screened for hub genes common with DEGs. To elucidate key biological functions and pathways related to PMOP, we conducted functional enrichment analyses using GO, KEGG, and GSEA. Additionally, we validated the expression and diagnostic values of hub genes in PMOP using external datasets GSE56814 and GSE147287. Furthermore, we assessed the immune infiltration levels in PMOP and investigated the correlation between hub genes and immune cell expression. Our pan-cancer analysis revealed the potential involvement of PMOP-related genes in cancer development, progression, and prognosis. This is the first study to apply a combined approach of WGCNA and machine learning to identify potential diagnostic and therapeutic markers for PMOP, explore the mechanism of its immune response involvement in pathogenesis, and evaluate the functional significance of candidate genes at the level of pan-cancer. Our findings offer a strong theoretical foundation for future development of novel gene or molecular therapies for PMOP and provide new insights into a potential association between PMOP and cancer.

## RESULTS

### Identification of the DEGs in PMOP

**Figure 2:**
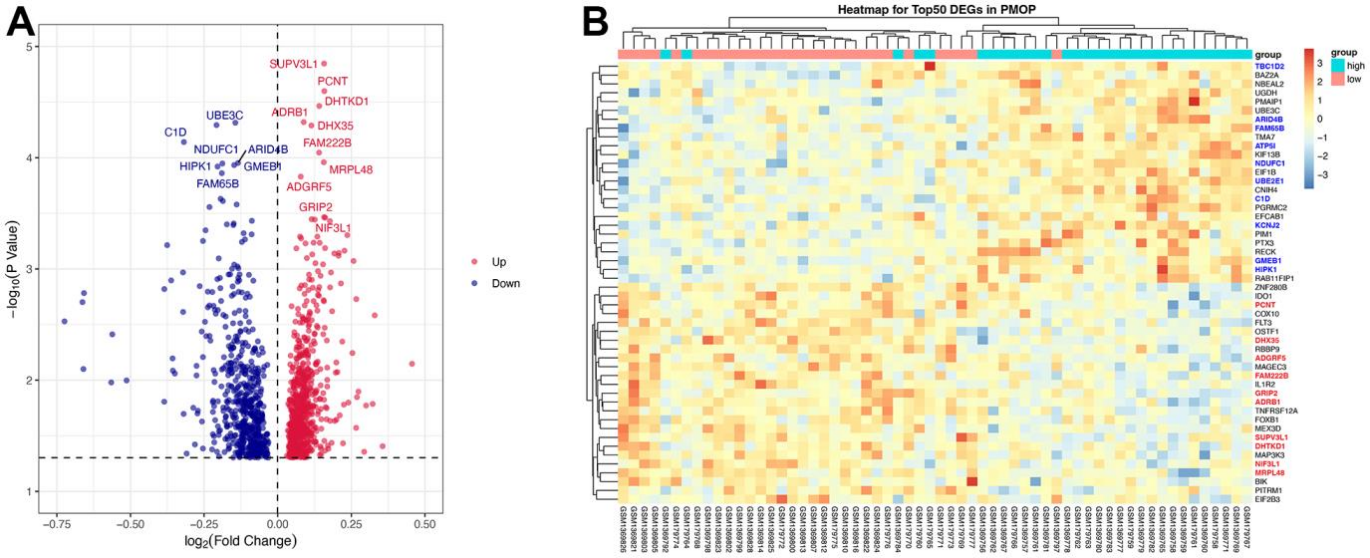
DEGs screening. Volcano plot (**A**) and heatmap (**B**) for the DEGs identified from the integrated PMOP dataset. Top regulated genes were texted in the volcano plot. The top 10 up-regulated genes are highlighted in bold red on the heatmap while the top 10 down-regulated genes are in bold blue.

After removing batch effects, mRNA expression levels in Peripheral blood mononuclear cells (PBMC) were obtained from PMOP patients and healthy controls in two GEO datasets (GSE56815 and GSE7429). The screening criteria of DEGs was |log2(fold change) |≥ 0 and adjusted p value< 0.05 [24]. Totally 1278 DEGs were identified with 715 up-regulated genes and 563 down-regulated genes. Sorted by the values of log2(fold change), the top 10 up-regulated genes were SUPV3L1, PCNT, DHTKD1, ADRB1, DHX35, FAM222B, MRPL48, ADGRF5, GRIP2, and NIF3L1 while the top 10 down-regulated genes were KCNJ2, TBC1D2, ATP5l, UBE3C, C1D, ARID4B, NDUFC1, GMEB1, HIPK1, and FAM65B. Volcano plot (Figure 2A) visualized the results. Both top 10 up-regulated and down-regulated genes are highlighted in the heatmap (Figure 2B).

### Functional enrichment analysis of DEGs

We next perform the functional enrichment analysis on the 1278 DEGs. The results shown by bar plots were sorted by p-value and dot plots were ranked based on the counts of enriched genes. GO enrichment analysis indicated that the top 10 biological enriched functions were “actin binding”, “nuclear receptor binding”, “RNA polymerase Il-specific DNA-binding transcription factor binding”, “DNA-binding transcription factor binding”, “ion channel regulator activity”, “protein serine kinase activity”, “G protein beta-subunit binding”, “channel regulator activity”, “nucleocytoplasmic carrier activity”, and “heme transmembrane transporter activity” (Supplementary Figure 1A, 1B). With KEGG enrichment analysis, the key enriched pathways for DEGs were “PI3K-Akt signaling pathway”, “Regulation of actin cytoskeleton”, “cAMP signaling pathway”, “Coronavirus disease – COVID-19”, “Tight junction”, “Transcriptional misregulation in cancer”, “Thyroid hormone signaling pathway”, “Alcoholic liver disease”, “Platelet activation”, and “Autophagy - animal” (Supplementary Figure 1C, 1D).

### Weighted gene co-expression network analysis and construction

**Figure 3:**
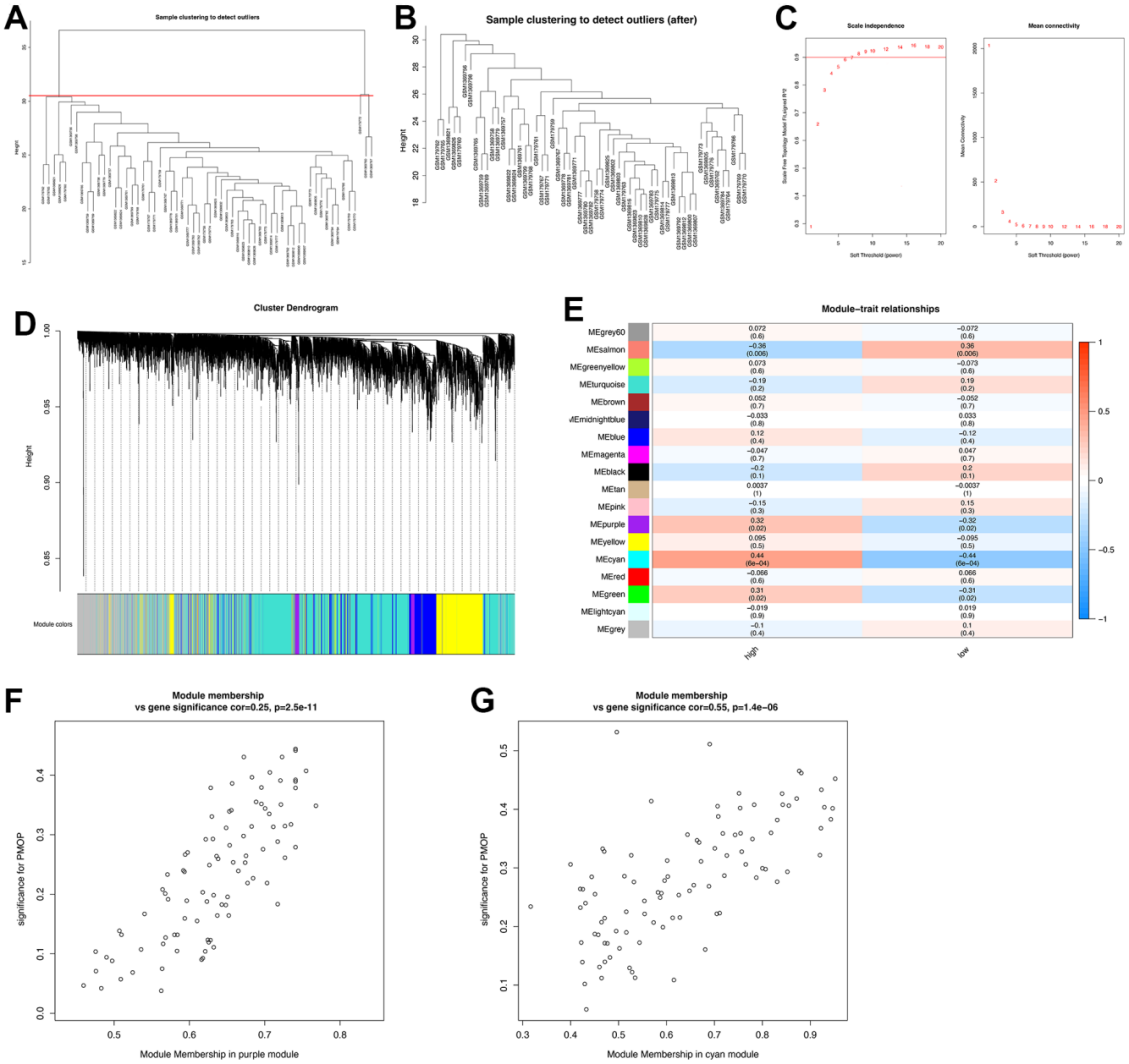
**Construction of WGCNA co-expression network.** (**A**) Sample clustering dendrogram and the samples whose height > 31 were identified as outliers. (**B**) Sample clustering dendrogram after cutting the outliers. (**C**) Soft threshold b = 7 and scale-free topological fit index (R2). (**D**) Shows the modules with different colors under the clustering tree. (**E**) Heat map of module-trait correlations. (**F**) MM vs. GS scatter plot of the purple module. (**G**) MM vs. GS scatter plot of the cyan module. Red represents positive correlations and blue represents negative correlations.

A total of 58 samples (30 PMOP patients and 28 healthy controls) were included in the WGCNA after clustering and excluding two outliers with the cut-off value =31 (Figure 3A, 3B). Depending on the curve of scale independence, the soft threshold was set to 7 when R2 >0.9 and the mean connectivity remained low (Figure 3C). Cluster Dendrogram was constructed, and 18 modules were identified with different features (Figure 3D). Then we explore the relationship between modules and clinical groups (high BMD and low BMD) to find the key module genes that may correlate greatly with PMOP. As shown in Figure 3E, the salmon module had a positive association with PMOP (r =0.36, p= 0.006) while the purple module (r =-0.32, p= 0.02), the cyan module (r= -0.44, p= 6E-04), and the green module (r= -0.31, p= 0.02) were negatively correlated with PMOP. Among the four modules, the purple module (cor=0.25, p=2.5e-11) and cyan module (cor=0.55, p=1.4e-06) showed a great correlation with PMOP in the MM versus GS scatterplot (Figure 3F, 3G). A total of 522 genes in these two modules were selected for further analysis.

### Functional enrichment analysis of the common genes

**Figure 4:**
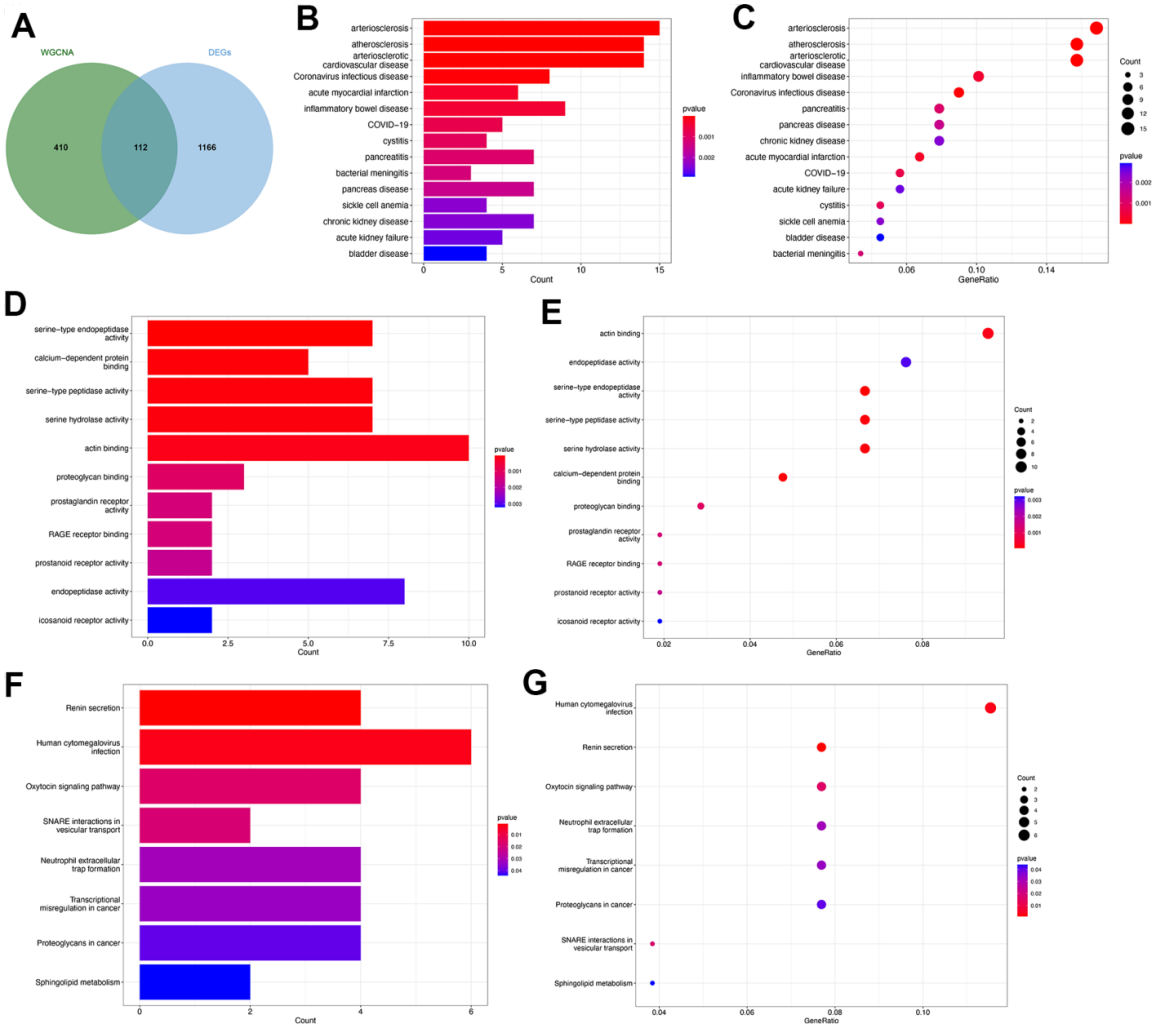
**Functional enrichment analysis of the common key genes.** (**A**) The Venn plot identified 112 shared genes among 522 module genes and 1278 DEGs. Bar plot (**B**) and dot plot (**C**) showed the results of DO enrichment analysis of 112 common genes. Bar plot (**D**) and dot plot (**E**) showed the results of GO enrichment analysis of 112 common genes. Bar plot (**F**) and dot plot (**G**) showed the results of KEGG enrichment analysis of 112 common genes.

**Figure 5:**
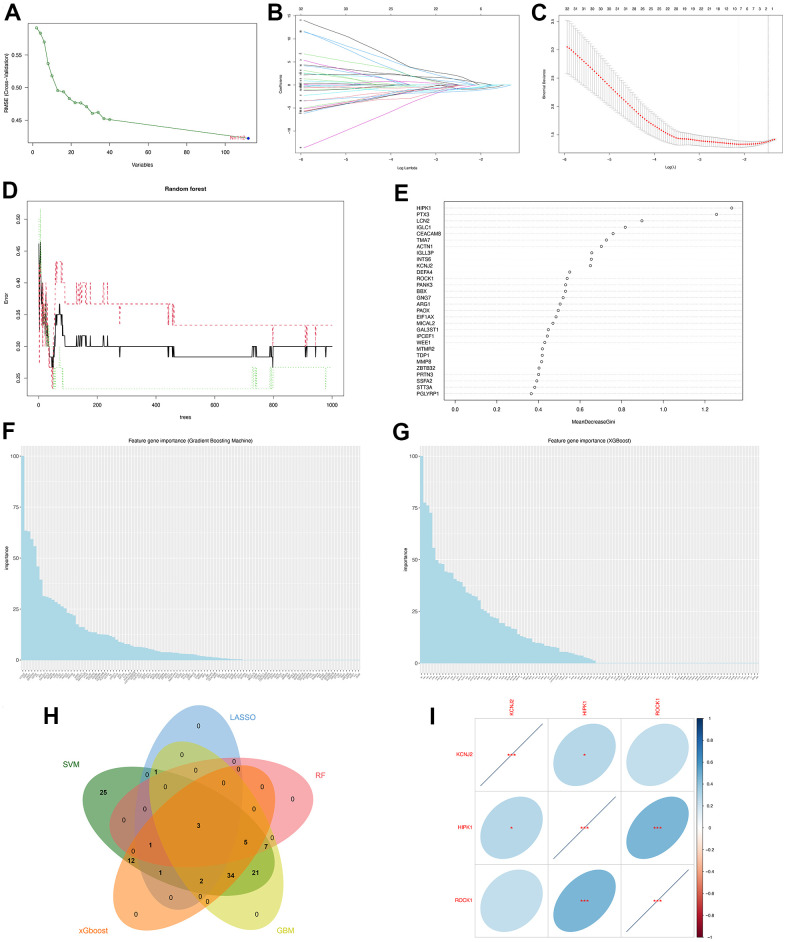
**Machine learning identified three hub genes of PMOP.** (**A**) SVM-RFE screening of candidate diagnostic genes. (**B**, **C**) LASSO screening of candidate diagnostic genes. (**D**) Random forest error rate versus the number of classification trees. (**E**) Random forest calculated the top 30 relatively important genes of PMOP. (**F**) GBM screening of candidate diagnostic genes and the bar chart showed the genes ranked by importance. (**G**) XGboost screening of candidate diagnostic genes and he bar chart showed the genes ranked by importance. (**H**) Venn plot between five machine learning methods resulted in three common hub genes. (**I**) Correlation between three hub genes. Blue represents positive correlations and red represents negative correlations. *, p < 0.05, **, p < 0.01, ***, p < 0.001.

The genes identified through the employment of limma and WGCNA analyses have potential significance in the pathogenesis of PMOP. Subsequently, an intersection of the genes derived from both methods was conducted to identify key genes that exhibit consistent role in PMOP for the further analyses. We collected 522 genes from the key modules identified by WCGNA and Venn plot show a total of 112 common genes between 522 module genes and 1278 DEGs (Figure 4A). DO enrichment analysis showed the possible common genes-related diseases, including “arteriosclerosis”, “atherosclerosis”, “arteriosclerotic cardiovascular disease”, “acute myocardial infarction”, “inflammatory bowel disease”, “COVID-19”, “cystitis”, “pancreatitis”, and “bacterial meningitis” (Figure 4B, 4C). We also perform GO and KEGG functional enrichment analyses. The top five biological processes were “serine-type endopeptidase activity”, “calcium-dependent protein binding”, “serine-type peptidase activity”, “serine hydrolase activity”, and “actin binding” (Figure 4D, 4E). Pathways highly related to the 112 common genes included “Renin secretion”, “Human cytomegalovirus infection”, “Oxytocin signaling pathway”, “SNARE interactions in vesicular transport”, “Neutrophil extracellular trap formation”, “Transcriptional misregulation in cancer”, “Proteoglycans in cancer”, and “Sphingolipid metabolism” (Figure 4F, 4G). The results of GSEA for 112 common genes were visualized by ridge plots in Supplementary Figure 2A, 2B. Top three GO Cell Component (CC) contained “immunoglobulin complex circulating”, “secretory granule membrane”, and “vesicle lumen” and top three Biological Process (BP) included “respond to bacterium”, “response to molecule of bacterial origin”, and “innate immune response in mucosa” (Supplementary Figure 2A). KEGG pathway analysis by GSEA resulted in “alanine aspartate and glutamate metabolism”, “one carbon pool by folate”, and “chemokine signaling pathway” (Supplementary Figure 2B). Supplementary Figure 2C and Figure 5D were GSEA plots showing the top- and down-regulated GO terms respectively. For KEGG enrichment analysis, the top- and down-regulated pathways were also visualized by the GSEA plot (Supplementary Figure 2E, 2F).

### Screen of the hub genes by machine learning

Five machine-learning algorithms were used to identify the hub genes. SVM-RFE screened 112 PMOP-related feature genes (Figure 5A). LASSO coefficient spectrum (Figure 5B) and coefficient profile (Figure 5C) show the results of LASSO regression analysis that 8 hub genes were selected. Figure 5D shows how the RandomForest algorithm correlates the mistake rate with the number of classification trees. The relative importance of 30 genes was sorted for choosing the hub genes (Figure 5E), and 16 feature genes were identified. GBM (Figure 5F) and xGBoost (Figure 5G) algorithms sorted the 112 genes by feature gene importance while 73 and 58 hub genes were screened respectively. We used a Venn plot to find the common genes between the five machine-learning methods and three hub genes (KCNJ2, HIPK2, ROCK1) were identified for further study (Figure 5H). With the hub gene expression values in PMOP, HIPK1 was positively correlated with KCNJ2 (p <0.05) and ROCK1 (p <0.001) (Figure 5I).

### Protein-protein interaction network construction

**Figure 6:**
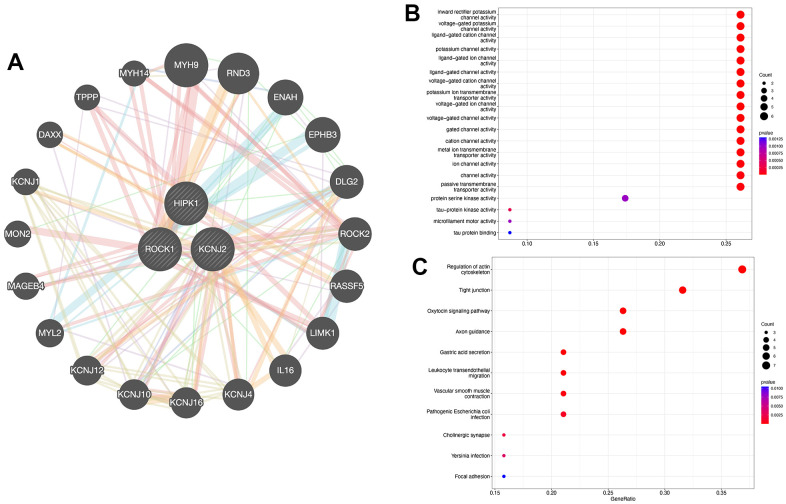
**PPI network construction.** (**A**) Interaction analysis of hub genes and the construction of gene co-expression network. (**B**) Dot plot showed the results of GO enrichment analysis of three hub genes and 20 related genes. (**C**) Dot plot showed the results of KEGG enrichment analysis of three hub genes and 20 related genes.

Figure 6A showed the interaction between 3 hub genes and a total of 20 highly related genes. Then we perform GO and KEGG analysis to further explore the GO terms and KEGG pathways which were enriched by the 23 genes. GO functional enrichment analysis mostly focuses on the channel activity, including “Inward rectifier potassium channel activity”, “Voltage-gated potassium channel activity”, and “Ligand-gated cation channel activity” (Figure 6B). As shown in Figure 6C, “Regulation of actin cytoskeleton”, “Tight junction”, “Oxytocin signaling pathway”, and “Axon guidance” were identified as the enriched pathways of the hub genes.

### Validation of the expression levels and the diagnostic values of the hub genes

**Figure 7:**
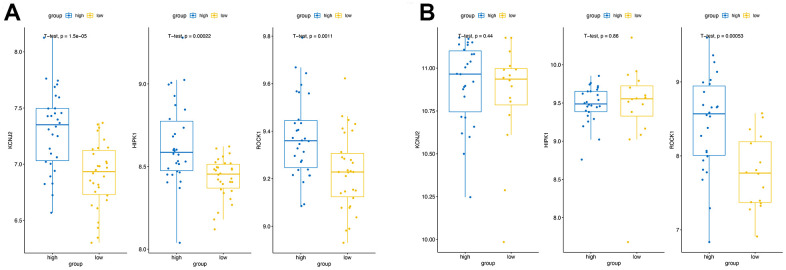
**Validation of the hub genes expression.** The expression of three hub genes in high BMD group and low BMD (PMOP) group of the training datasets (**A**) and the validation datasets (**B**).

**Figure 8:**
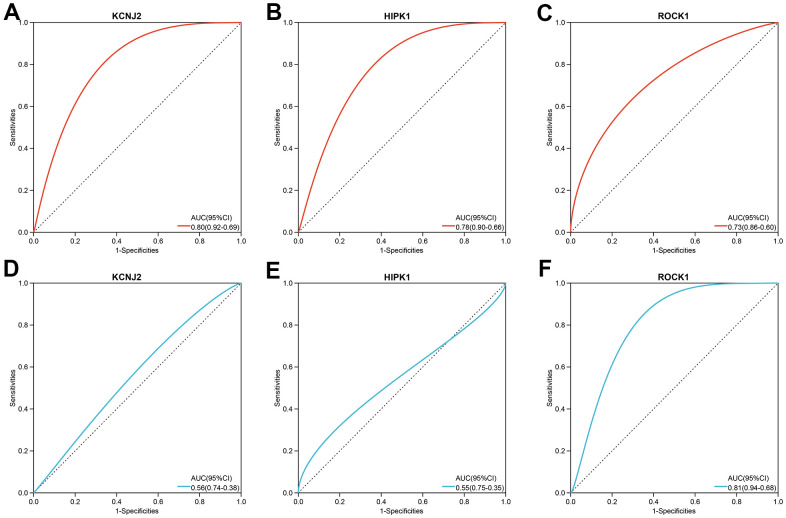
The three hub genes’ diagnostic value in PMOP training datasets (**A**–**C**) and validation datasets (**D**–**F**). ROC curves and AUC statistics are used to evaluate the capacity to discriminate PMOP from healthy controls with excellent sensitivity and specificity.

In the combined training datasets of PMOP (GSE56815 and GSE7429), the expression levels of KCNJ2, HIPK1, and ROCK1 were significantly lower in low BMD group (PMOP) than high BMD group (healthy controls) (p =1.5E-05, 2.2E-04, and 1.1E-03, respectively) (Figure 7A). We further downloaded the GSE56814 as the validation dataset, microarray analyses of monocytes from postmenopausal females with low or high BMD. Only ROCK1 showed decreased expression in PMOP group compared with controls (p =5.3E-04) (Figure 7B). Then we performed ROC analysis and calculated the AUC values to identify the diagnostic value of the hub genes. KCNJ2, HIPK1, and ROCK1 all have great abilities to diagnose PMOP in the training dataset with the AUC value >0.7 (AUC =0.80, 0.78, and 0.73, respectively) (Figure 8A–8C). However, like the same result as the expression data, the AUC value of ROCK1 was 0.81 while both KCNJ2 and HIPK1 have no significant value on PMOP diagnosis (Figure 8D–8F).

### Analysis of the role of ROCK1 in PMOP using GSEA

**Figure 9:**
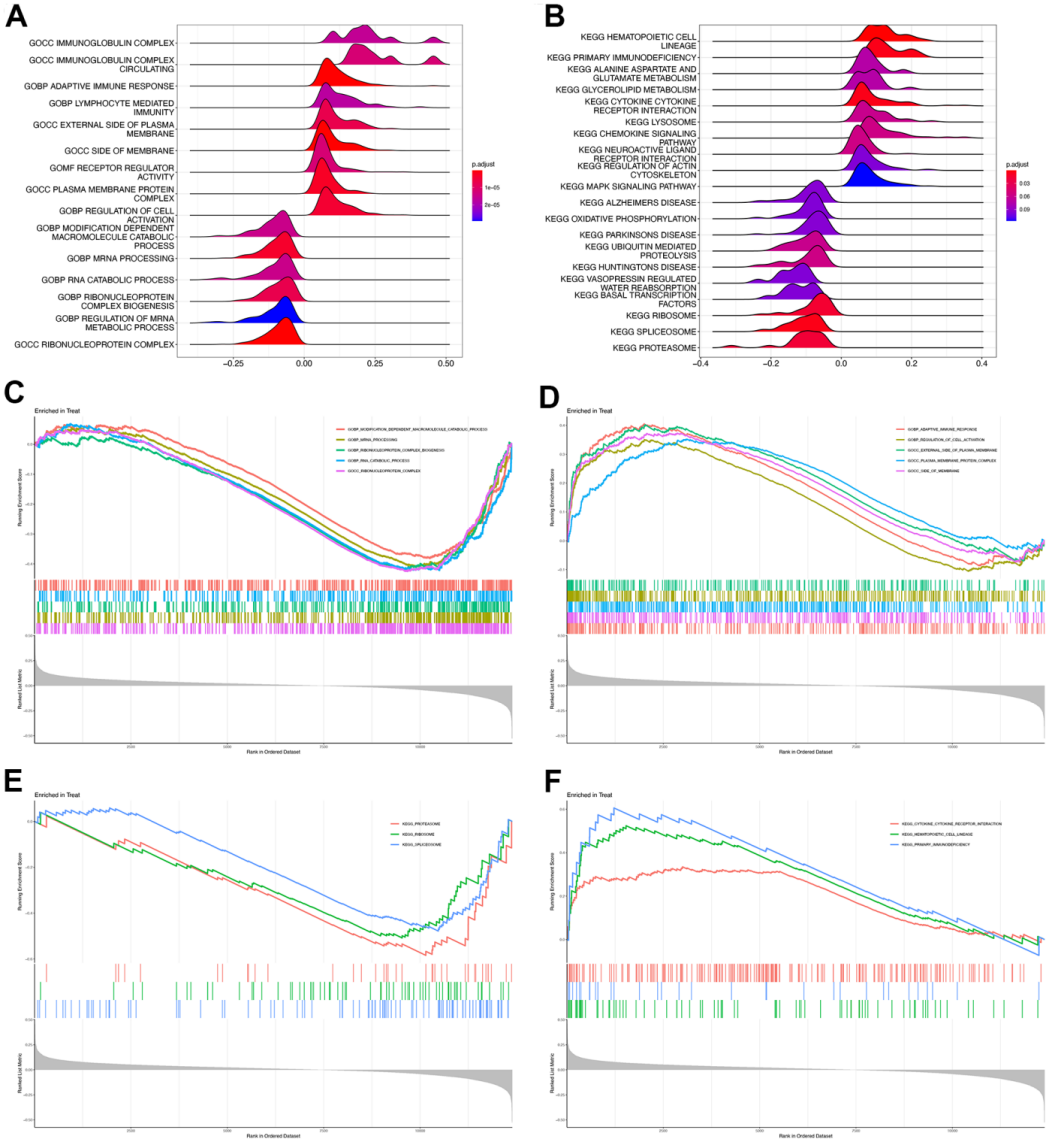
**GSEA of high and low ROCK1 subgroup.** Ridge map showed the GO (**A**) and KEGG (**B**) enrichment analysis results of all DEGs by GSEA. GSEA plot with DEGs and the top five GO terms enriched in high ROCK1 (**C**) and low ROCK1 (**D**) subgroup. GSEA plot with DEGs and the top three KEGG terms enriched in high ROCK1 (**E**) and low ROCK1 (**F**) subgroup.

Since ROCK1 had reduced expression and brilliant diagnostic ability in PMOP, we additionally divide the training dataset into two categories (high-ROCK1 group, and low-ROCK1 group) by the median values of the gene expression among the population to further identify the possible role of ROCK1 in PMOP. We first set GSEA for all the gene expression data of 30 healthy controls and 30 PMOP patients. GO enrichment analysis identified “immunoglobulin complex”, “immunoglobulin complex circulating”, “adaptive immune response”, and “lymphocyte-mediated immunity” as the top terms related to PMOP (Figure 9A). For KEGG analysis, the top enriched pathways were “hematopoietic cell lineage”, “primary immunodeficiency”, “alanine aspartate and glutamate metabolism”, “glycolipid metabolism”, and “cytokine receptor interaction” (Figure 9B). The genes of samples in the high-ROCK1 group enriched in “modification dependent macromolecule catabolic process”, “mRNA processing”, “ribonucleoprotein complex biogenesis”, “RNA catabolic process”, and “ribonucleoprotein complex” (Figure 9C). “proteasome”, “ribosome”, and “spliceosome” were the top three results analyzed by KEGG (Figure 9E). The genes in the low-ROCK1 group may have a positive association with PMOP, and the enriched biological terms were “adaptive immune response”, “regulation of cell activation”, “external side of plasma membrane”, “plasma membrane protein complex”, and “side of membrane” (Figure 9D). KEGG functional enrichment analysis indicated that “cytokine receptor interaction”, “hematopoietic cell lineage”, and “primary immunodeficiency” were greatly correlated with samples of the low-ROCK1 group (Figure 9F).

### Immune infiltration analysis and the correlation between genes and immune

**Figure 10:**
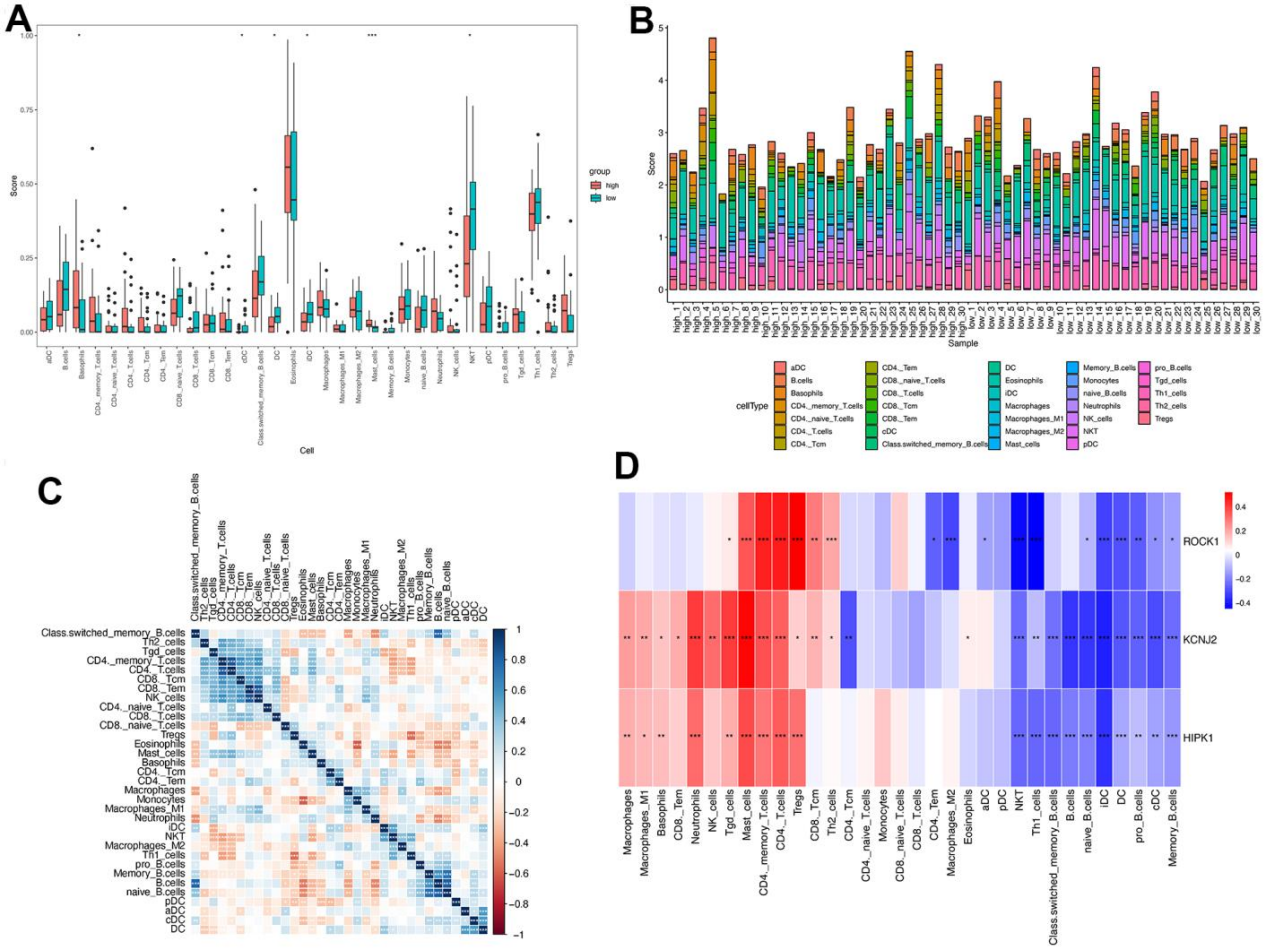
**Immune cell infiltration analysis on PMOP.** The proportion of 33 kinds of immune cells in PMOP patients and controls was visualized from the box plot (**A**) and the bar plot (**B**). (**C**) Correlation of 33 immune cell type compositions in PMOP. (**D**) Correlation between the expression of 33 immune cells and three hub genes in PMOP. *, p < 0.05, **, p < 0.01, ***, p < 0.001.

According to the above GSEA results for 112 overlapped genes, three hub genes, and low-ROCK1 group genes, both the GO and KEGG functional enrichment analyses showed a probable association between immunization and PMOP, such as “innate immune response in mucosa”, “adaptive immune response”, “lymphocyte-mediated immunity”, and “primary immunodeficiency”. We then perform an immune infiltration analysis by xCell to find the content and effect of 33 immune cell types on PMOP. As shown in Figure 10A, DCs (Dendritic cells), cDCs (conventional DCs), iDCs (immature DCs), and NKT (Natural Killer T) cells were elevated (p <0.05) while Basophils and Mast cells show a significant decrease (p <0.05 and <0.001, respectively) in PMOP patients. Bar plot showed the different compositions and immune scores of immune cells in each sample (Figure 10B). The correlations between the expression levels of 33 immune cells were calculated. DC had a negative relationship with the T cells family, including Th2 cells, Tgd cells, CD4+ T cells, and CD8+ T cells (Figure 10C). For NKT cells, it was inversely related to Th2 cells but positively related to Th1 cells and Macrophages M2 (Figure 10C). Basophils and Mast cells have a positive correlation with Monocytes and B cells while Mast cells also had a positive association with T cells and Neutrophils (Figure 10C). The correlation between three hub genes and immune cells expression was shown in Figure 10D, and ROCK1 was inversely related to CD4+ TEM (Effector memory T Cells), Macrophages M2, aDC, cDC, iDC, DC, NKT, Th1 cells, naïve B cells, pro B cells, and memory B cells. Conversely, Tgd cells, Mast cells, CD4+ memory T cells, CD4+ T cells, Tregs, CD8+ Tcm, and Th2 cells were negatively correlated with ROCK1 in PMOP patients (Figure 10D).

### Single-cell analysis

**Figure 11:**
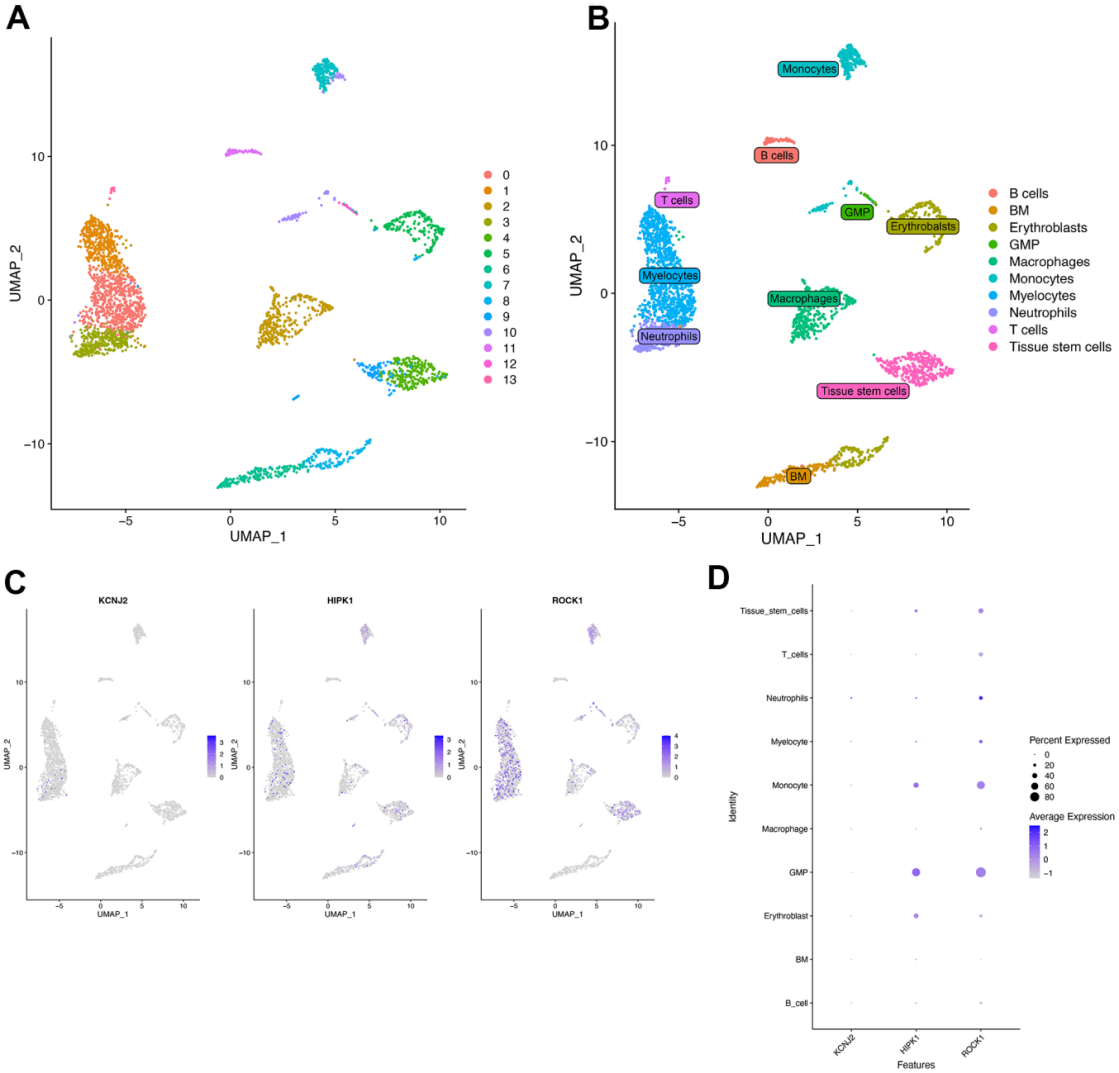
**Single-cell RNA analysis on bone marrow-derived mesenchymal stem cells (BM-MNCs) from PMOP patients.** (**A**) UMAP plot showed 13 clusters of BM-MNCs from PMOP patients. (**B**) UMAP distributions of single cells from the 10 defined cell types annotated by SingleR. Feature plot (**C**) and dot plot (**D**) showed the expression of three hub genes in identified clusters and cell types.

The single-cell RNA sequencing data was from a 67-year-old postmenopausal woman’s bone marrow derived mononuclear cells (BM-MNCs). The UMAP (Figure 11A) showed the 13 clusters of whole cells after dimension and clustering. We then used the “SingleR” package to annotate the 13 clusters into biological cell types, and the 13 clusters were B cells, BM (Bone Marrow), Erythroblasts, GMP (Ganulocyte-Monocyte Progenitor), Macrophages, Monocytes, Myelocytes, Neutrophils, T cells, and Tissue stem cells (Figure 11B). The distribution of three hub genes (KCNJ2, HIPK1, ROCK1) in each cluster was shown in Figure 11C by feature plot. As shown in Figure 11D, ROCK1 was highly expressed in Neutrophils, and the percent expressed of ROCK1 seemed more in GMP and Monocytes.

### Pan-cancer ROCK1 expression

Several studies have found the important role of ROCK1 in cancers [25–28]. Since PMOP patients had a higher risk to get some types of cancer, we were greatly interested in whether the low expression of ROCK1 made great importance to increasing the cancer risk, accelerating the cancer progression, or worsening the cancer prognosis [29, 30].

**Figure 12:**
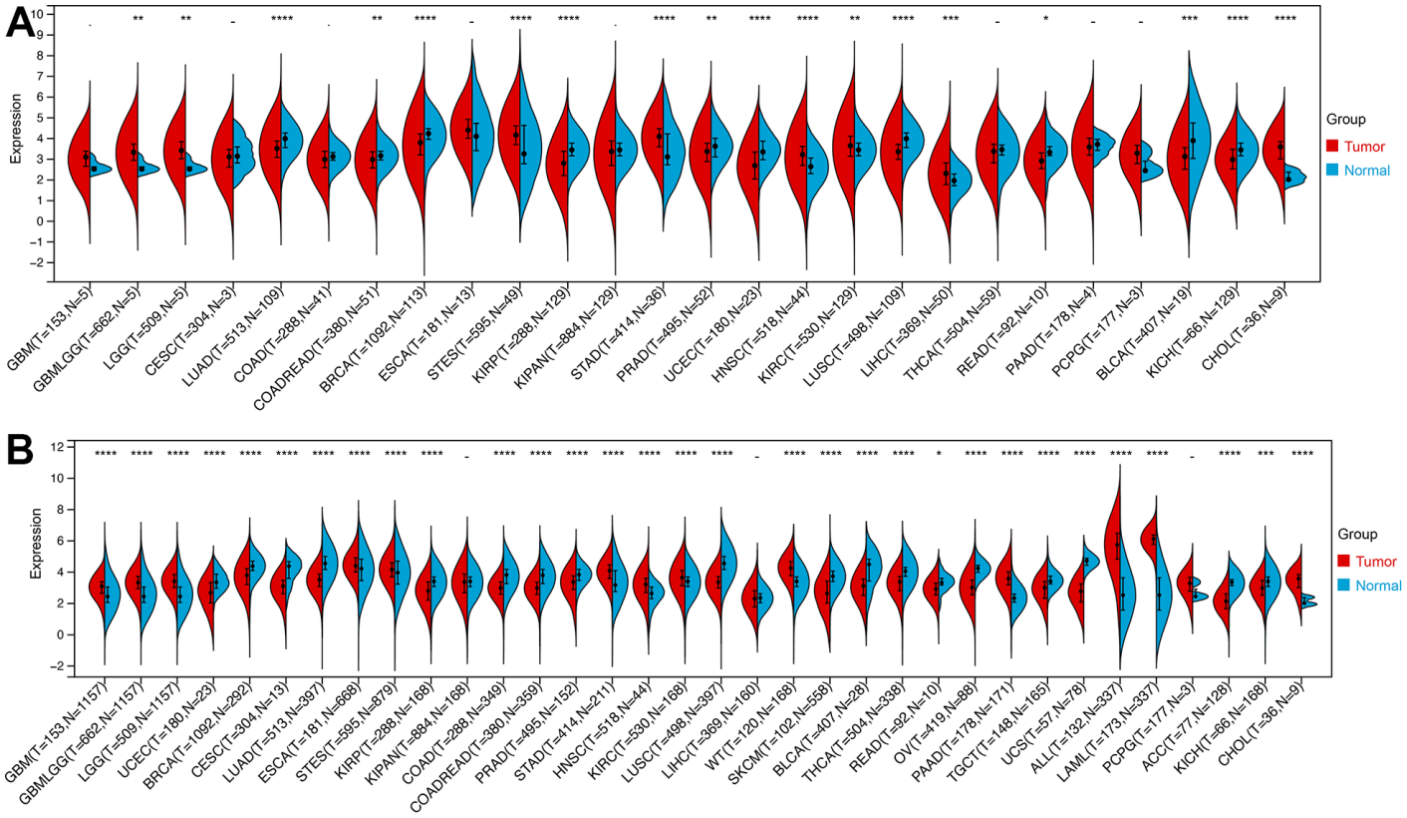
**The expression of ROCK1 in Pan-cancer.** (**A**) Pan-cancer expression levels of ROCK1 in the TCGA dataset. (**B**) Pan-cancer expression levels of ROCK1 in the TCGA and GTEx datasets. *p <0.05, **p <0.01, ***p <0.001, ****p <0.0001, -no significance.

**Figure 13:**
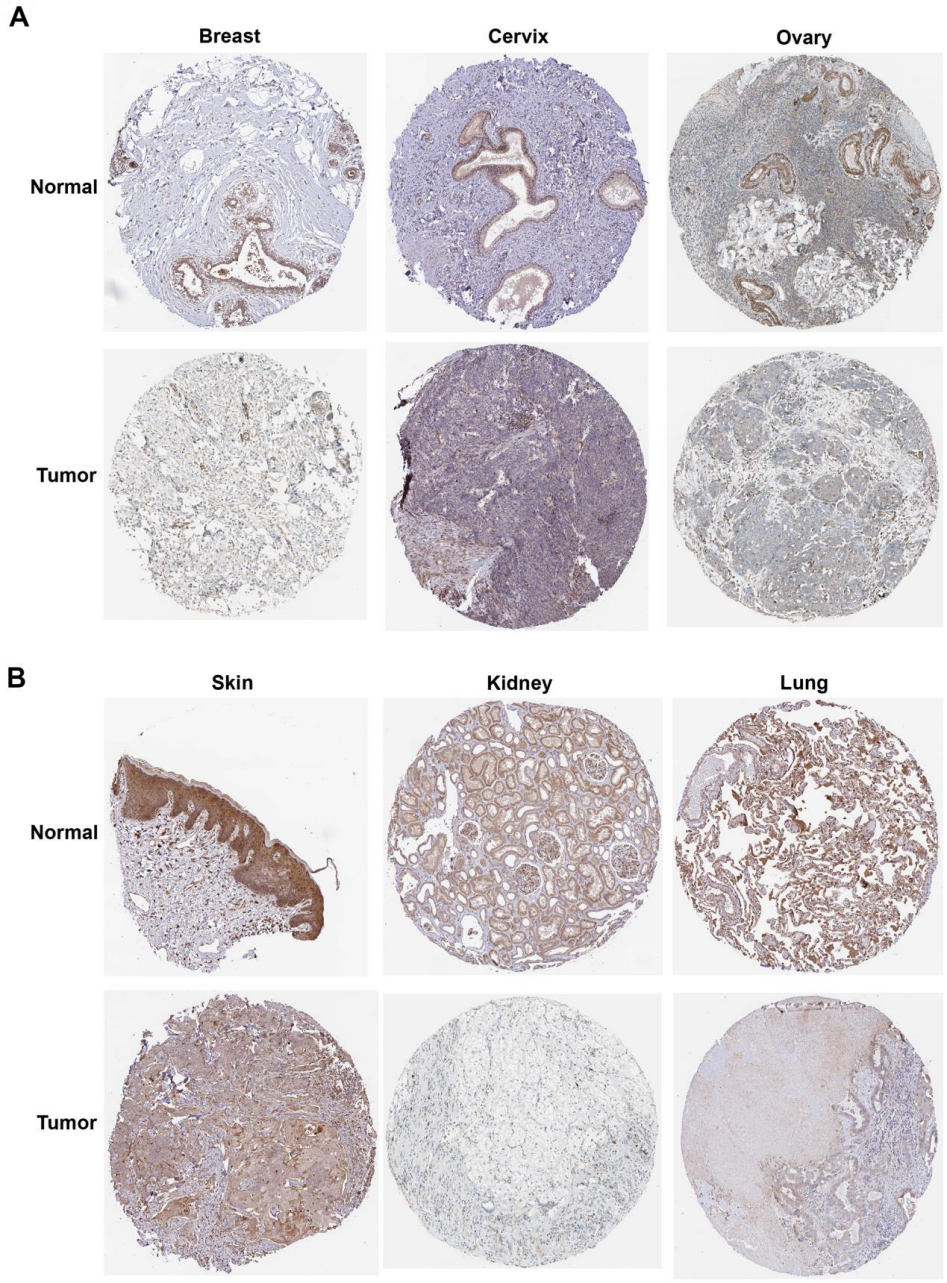
**The protein expression of ROCK1 in pan-cancer.** (**A**) The protein expression level of ROCK1 in women-specific cancers (Breast tumors, Cervical tumors, and Ovarian tumors versus normal tissues. (**B**) The protein expression level of ROCK1 in Skin tumors, Renal tumors, and Lung tumors versus normal tissues.

We then calculated the difference in expression between normal and tumor samples in each tumor. In TCGA datasets of 26 tumors, eight tumors showed significantly higher expression, including Glioma (GBMLGG), Brain Lower Grade Glioma (LGG), Stomach and Esophageal carcinoma (STES), Stomach adenocarcinoma (STAD), Head and Neck squamous cell carcinoma (HNSC), Kidney renal clear cell carcinoma (KIRC), Liver hepatocellular carcinoma (LIHC), and Cholangiocarcinoma (CHOL), while the expression of ROCK1 in 10 tumors was lower than normal controls: Lung adenocarcinoma (LUAD), Colon adenocarcinoma/Rectum adenocarcinoma Esophageal carcinoma (COADREAD), Breast invasive carcinoma (BRCA), Kidney renal papillary cell carcinoma (KIRP), Prostate adenocarcinoma (PRAD), Uterine Corpus Endometrial Carcinoma (UCEC), Lung squamous cell carcinoma (LUSC), Rectum adenocarcinoma (READ), Bladder Urothelial Carcinoma (BLCA), and Kidney Chromophobe (KICH) (Figure 12A). To further validate the results, we add normal control data from GTEx datasets and perform difference analysis combined with TCGA and GTEx. As shown in Figure 12B, ROCK1 in 13 types of tumors was highly expressed: GBM(p=4.8E-12), GBMLGG(p=1.3E-73), LGG(p=1.5E-74), Esophageal carcinoma (ESCA)(p=2.9E-05), STES(p=4.0E-05), STAD(p=8.0E-22), HNSC(p=2.8E-07), KIRC(p=2.6E-06), High-Risk Wilms Tumor (WT)(p=5.1E-22), Pancreatic adenocarcinoma (PAAD)(p=7.7E-40), Acute Lymphoblastic Leukemia (ALL)(p=9.1E-44), Acute Myeloid Leukemia (LAML)(p=3.1E-74), CHOL(p=1.6E-05), and totally 18 cancers were low-ROCK1 expression: UCEC(p=5.1E-05), BRCA(p=3.0E-46), Cervical squamous cell carcinoma and endocervical adenocarcinoma (CESC)(p=7.7E-06), LUAD(p=8.4E-90), KIRP(p=2.7E-15), Colon adenocarcinoma (COAD)(6.8E-40), COADREAD(p=7.9E-47), PRAD(p=4.3E-16), LUSC(p=3.5E-103), Skin Cutaneous Melanoma (SKCM)(p=7.3E-24), BLCA(p=3.1E-08), Thyroid carcinoma (THCA)(p=1.5E-56), READ(p=0.02), Ovarian serous cystadenocarcinoma (OV)(p=6.8E-40), Testicular Germ Cell Tumors (TGCT)(p=3.0E-15), Uterine Carcinosarcoma (UCS)(p=1.6E-22), Adrenocortical carcinoma (ACC)(p=3.0E-24), KICH(p=2.6E-04). According to the results, we found that the tumors in the central nervous system (GBM and LGG), in the digestive tract (STAD, LIHC, CHOL, ESCA, PAAD), and the hematological malignancies including ALL and LAML had a higher level of ROCK1 expression significantly. Interestingly, consistent with the low expression of ROCK1 in PMOP, ROCK1 was down-regulated in all women’s tumors (BRCA, UCEC, CESC, OV, and UCS) in the database. Moreover, lung cancers (LUAD, LUSC) and tumors in the large intestine (COADREAD, READ, and COAD) also had a lower expression of ROCK1 than the normal tissues. However, the ROCK1 level in bone-related tumors like osteosarcoma is similar to the normal tissues. To further validate the results in protein level, the protein expression level of ROCK1 from HPA was consistent with the mRNA results, both in women-specific cancers (Figure 13A) and the skin cancers, renal cancers, and lung cancers (Figure 13B) which were mentioned above. We selected patients who is woman and at the age of postmenopause, the details of patient information were shown in Supplementary Table 2.

We also calculated the variation in ROCK1 expression in each tumor at different clinical phases to assess the impact of ROCK1 on tumor progression and severity. For the T stage (Supplementary Figure 3A), we found a significant difference in 5 tumors: BRCA (p=0.04), STAD (p=1.1E-03), PRAD (p=0.02), KIRC (p=4.3E-03), TGCT (p=0.02). ROCK1 expression in two tumors, STAD (p=0.01) and THCA (p=1.2E-03) were significant differences between N stages (Supplementary Figure 3B). Among 25 tumors for analysis, only KIRC M1 stage had lower ROCK1 expression than M0 stage (p=7.2E-03, Supplementary Figure 3C). Moreover, ROCK1 expression among Stage I to IV was significantly different in STAD (p=2.4E-03) and KIRC(p=2.5E-03) (Supplementary Figure 3D), while KIPAN (p=9.8E-07), HNSC (p=0.04), and KIRC (p=9.8E-07) had different ROCK1 expressions among Grade I to IV (Supplementary Figure 3E).

**Figure 14:**
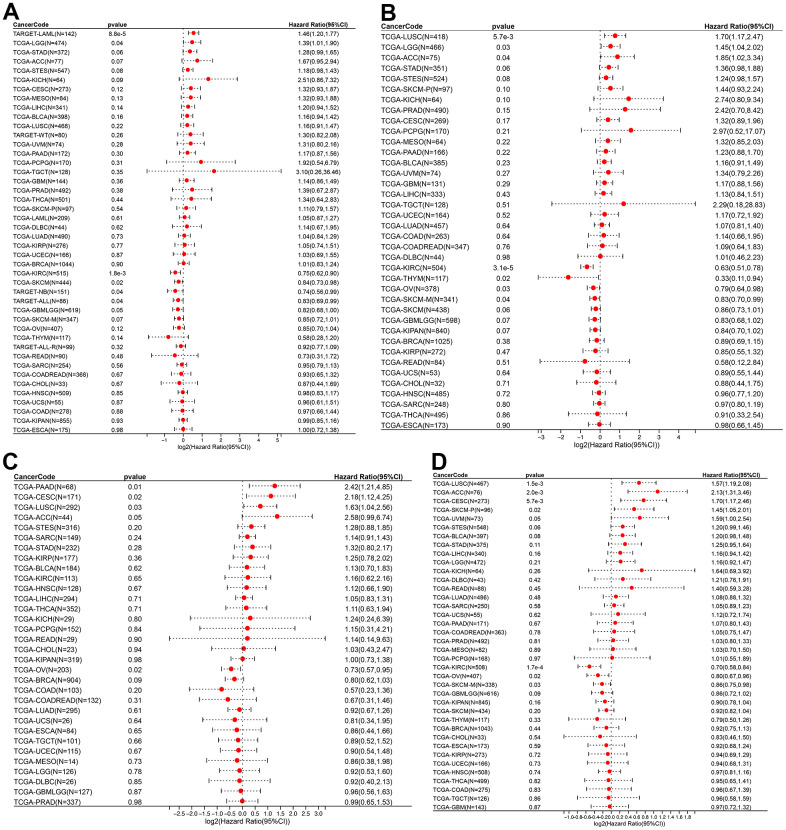
**ROCK1 and survival situations.** (**A**–**D**) Forest plots of ROCK1 expression and OS, DSS, DFI, and PFA. OS, overall survival; DSS, disease-specific survival; DFI, disease-free interval; PFI, progression-free interval; HR, hazard ratio.

The relationship between ROCK1 and tumor prognosis in pan-cancer should be explored, which may help further understand the role of low-ROCK1 expression in PMOP patients’ cancer risk. Among several pan-cancer, we calculated the survival time including Overall survival (OS), Disease-specific survival (DSS), Disease-free interval (DFI), and Progression-free interval (PFI). TCGA-LGG (HR= 1.39, 95%CI = 1.01-1.90), TARGET-LAML (HR =1.46, 95%CI =1.20-1.77) had lower OS time and worse prognosis with high ROCK1 expression (Figure 14A). In contrast, low expression of ROCK1 in TCGA-GBMLGG (HR= 0.82, 95%CI =0.68-1.00), TCGA-KIRC (HR= 0.75, 95%CI =0.62-0.90), TCGA-SKCM (HR= 0.84, 95%CI =0.73-0.98), TARGET-NB (HR =0.74, 95%CI =0.56-0.99), TARGET-ALL (HR= 0.83, 95%CI =0.69-0.99) was related to bad prognosis and high death risk (Figure 14A). TCGA-LGG, TCGA-LUSC, and TCGA-ACC had lower DSS time while the ROCK1 expression was higher, and lower DSS time was also connected to lower ROCK1 expression in TCGA-KIRC, TCGA-THYM, TCGA-SKCM-M, and TCGA-OV (Figure 14B). The data of DFI and PFI were also calculated and shown in Figure 14C, 14D. We then draw the Kaplan–Meier (K-M) curve of tumors which significantly affected the OS time and cancer prognosis, and further demonstrate that TCGA-GMBLGG, TCGA-KIRC, TCGA-SKCM, and TARGET-ALL had worse prognosis with low-ROCK1 expression while the high expression of ROCK1 in TCGA-LGG, TARGET-LAML, and TARGET-NB was correlated to the low survival probability (Supplementary Figure 4).

RNA modification, including m1A, m5C, and m6A, had been extensively investigated due to its significant association with both cancers and bone metabolism [31–34]. We further investigate the correlation between ROCK1 expression and RNA modification genes in pan-cancer. The 44 RNA modification genes for analysis included 10 m1A-related genes, 13 m5C-related genes, and 21 m6A-related genes, which were divided into writer, reader, and eraser. Most RNA modification gene expression was positively correlated to the ROCK1 (Supplementary Figure 5).

**Figure 15:**
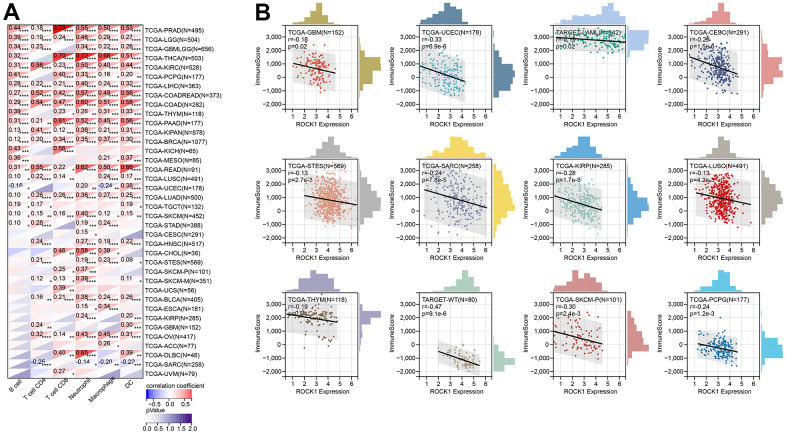
**ROCK1’s role in tumor immune response.** (**A**) Correlation between the expression levels of ROCK1 and immune infiltration pan-cancer by TIMER database. (**B**) Correlation between the expression of ROCK1 and ImmuneScore derived from the ESTIMATE algorithm. *p <0.05, **p <0.01, ***p <0.001, ****p <0.0001.

Using TIMER, six immune cell infiltration scores were obtained for 9406 tumor samples from 38 tumor types. Moreover, the immune infiltration score with a significant correlation was determined using Pearson’s correlation coefficient of gene and immune cell infiltration scores in each tumor. The full results were shown in Figure 15A. To further validate the TIMER results, we then perform ESTIMATE immune score analysis and find immune scores in 12 types of tumors (GBM, UCEC, LAM, CESC, STES, SARC, KIRP, LUSC, THYM, WT, SKCM-P, and PCPG) were negatively correlated to the ROCK1 expression levels (Figure 15B).

Tumor mutation burden (TMB) refers to the total number of substitution and insertion/deletion mutations per megabase in the exon coding region of a tumor sample that has been analyzed. High TMB levels in patients may result in increased neoantigen production and subsequent activation of tumor-specific T cells, which can be further potentiated by PD-1/PD-L1 inhibitors. As such, TMB has emerged as an important biomarker for predicting the response to immune checkpoint inhibitors in cancer patients [35]. We then calculate the Pearson correlation between TMB and ROCK1, which may help to identify ROCK1-related PD-1/PD-L1 inhibitors which can treat the specific tumors. As shown in Supplementary Figure 6A, ROCK1 expression had a positive correlation with TMB in COADREAD (r =0.12, p =0.026) and a negative correlation with STES (r =-0.16, p =1.7E-04) and STAD (r =-0.14, p =0.004). MSI (Microsatellite Instability) is a functional defect caused by DNA mismatch repair in tumor tissue. Like TMB, MSI is also an important tumor marker that may indicate a great effect treated by PD-1/PD-L1 inhibitors [36]. High ROCK1 expression was significantly and positively associated with high MSI values in GBM (r =0.23, p =0.005), GBMLGG (r =0.18, p =3.5E-06), CESC (r =0.15, p =0.01), LUAD (r =0.096, p =0.03), COAD (r =0.17, p =0.004), COADREAD (r =0.17, p =7.5E-04), LUSC (r =0.13, p =0.005), and READ (r =0.28, p =0.008), and with low MSI values in BRCA (r =-0.1, p =0.001), STES (r =-0.14, p =7.2E-04), KIPAN (r =-0.09, p =0.01), STAD (r =-0.12, p =0.016), PRAD (r =-0.16, p =3.2E-04), HNSC (r =-0.24, p =4.5E-08), THCA (r =-0.1, p =0.028), and DLBC (r =-0.56, p =3.8E-05) (Supplementary Figure 6B).

## DISCUSSION

Osteoporosis, a systemic skeletal disorder characterized by low bone mass and degradation of bone tissue microstructure, is a leading cause of bone fragility and susceptibility to fractures. Postmenopausal osteoporosis (PMOP) is the most common type of primary osteoporosis, affecting over 200 million women globally, and influenced by a range of factors, including age, smoking, drug use, dietary habits, physical activity, endocrine status, comorbidities, and genetics [11, 37]. A survey from Hong Kong found that women over the age of 50 had a prevalence of PMOP in the spine of 34.1-37%, compared to only 7% in men of the same age, indicating that low estrogen levels in women may increase their risk [38]. PMOP results in decreased bone mass, changes in bone tissue composition, increased fragility, and susceptibility to fractures, as well as pain, bone deformities, and related complications, all of which have a significant negative impact on the health and quality of life of elderly women and may even reduce their lifespan [39]. For individuals at low risk of fractures, the North American Menopause Society (NAMS) recommends non-pharmacologic interventions, including healthy eating (with adequate protein, calcium, and vitamin D), regular physical activity, and avoidance of smoking and excessive alcohol consumption [40, 41]. Bisphosphonates are the most commonly used drug for PMOP; however, they can have harmful effects, such as osteonecrosis of the jaw, renal impairment, atypical femur fractures, upper gastrointestinal side effects, and atrial fibrillation [42–46]. To date, several genome-wide association studies (GWASs) have found hundreds of candidate genes associated with osteoporosis, including RANKL, SOST, ESR1, PTHLH, and DKK1 [47]. Targeting these genes with RANKL inhibitors, sclerostin inhibitors, estrogen agonists, parathyroid hormone-receptor agonists, and DKK1 inhibitors has been partially approved for clinical use in high-risk patients requiring pharmacologic interventions [47, 48]. Therefore, exploring novel genomic targets can provide further and innovative perspectives on PMOP therapeutic approaches.

With the rapid development of computer science, bioinformatics was the study of large biological data, including DNA, RNA, and protein sequences, which is crucial for the detection and treatment of disease. We can distinguish the traits of diseases at the genetic level thanks to newly developed high-throughput techniques like gene microarray chips, RNA sequencing, and scRNA sequencing. Moreover, WGCNA and Machine Learning can screen out characteristic genes that are highly associated with disease phenotypes through computer algorithms such as clustering, iteration, training, and sorting. These methods enable quick and accurate early detection of diseases and the development of highly effective treatments by targeting specific genes.

Getting access to the data from RNA-seq or microarray data, previous studies have illustrated the role of hub genes in PMOP by WGCNA or PPI network analysis [49–51]. However, there is still a paucity of research on identifying the hub genes by performing machine learning combined with WGCNA on mRNA as well as scRNA expression data. In the study, we identified the 715 up-regulated and 563 down-regulated DEGs in PMOP patients from a training mRNA dataset and screened the hub genes using WGCNA and Machine Learning. Finally, three hub genes (KCNJ2, HIPK1, and ROCK1) were identified. Functional enrichment analyses, including DO, GO, KEGG, and GSEA, were performed on DEGs and the hub genes. The binding between proteins, ion channel activity, and immune reaction activity were three types of enrichment results that keep occurring. PPI co-expression network further illustrated the interaction between the hub genes which may influence the development of PMOP. The expression and diagnostic values of the hub genes were validated in the external mRNA dataset and finally, ROCK1 showed significantly low expression (p =5.3E-04) and a high diagnostic value (AUC =0.81) in PMOP, further illustrating its potential key role in PMOP.

KCNJ2 is a member of the KCNJ genes family which encodes inwardly-rectifying potassium (Kir) channels [52]. The mutation of KCNJ2 was widely reported in Andersen’s Syndrome, characterized by recurrent paralysis, irregular heartbeat, and defects in bone growth [53]. Moreover, a mutation in 17q24.3-rs12946942 near the KCNJ2 may be associated with the phenotypes of adolescent idiopathic scoliosis [54]. Only one GWAS study on a total of 5428 Chinese population has found rs1239055408 G>GA (KCNJ2) was associated with BMD only in women, and 17q24.3-rs1239055408 had significantly stronger effects in women [55]. Further research should focus on the molecular mechanism of KCNJ2 in the pathogenesis of PMOP. The homeodomain-interacting protein kinase (HIPK) family consists of four closely related serine/threonine kinases, HIPK1-4, that have been found as co-repressors for homeodomain-containing transcription factors [56]. HIPK plays an important role in maintaining normal body development and diseases such as tumors, fibrosis, and tissue malformation [57]. However, no study has explored the relationship between HIPK1 and PMOP to date. The deletion of HIPK in the mouse model impaired glucose tolerance and insulin secretion, leading to a higher risk of type 2 diabetes [58]. Since patients with type 2 diabetes had impaired metabolism and a higher prevalence of osteoporosis in postmenopausal women, whether the low expression of HIPK1 impairs the body’s overall metabolic capacity and leads to PMOP needs to be further investigated and validated [59–61]. Rho-associated kinase ROCK1 was downstream of Rho GTPase that regulates the actin cytoskeleton dynamics. The variants of ROCK1 were associated with disease susceptibilities like cardiovascular diseases, cancers, autoimmune diseases, and glaucoma [26]. It has been reported that Hsa_circ_0006859 is the competing endogenous RNA of miR-431-5p that increases the expression of ROCK1 in human bone marrow mesenchymal stem cells (hBMSCs) from PMOP patients, and the capacity of hBMSCs to differentiate was predisposed from osteogenesis towards adipogenesis [62]. Studies conducted *in vitro* have shown that endoplasmic reticulum stress and autophagy failure cause inflammatory bone loss by activating the ROCK1 signaling pathway in BMSCs [63]. Conversely, by increasing ROCK1 expression, strontium ranelate (SrR, an anti-osteoporosis drug) promotes the osteogenesis of ovariectomy rat bone marrow mesenchymal stem cells (OVX-rBMSCs) and cell viability of primary osteoblasts [64]. These inverse results indicate that more studies should be performed to identify the role of ROCK1 in PMOP.

We then identified ROCK1 as the key gene for further analysis. Divided the PMOP patients into two groups based on the expression of ROCK1, the GSEA analysis illustrated that “adaptive immune response”, “regulation of cell activation”, “external side of plasma membrane”, “plasma membrane protein complex”, and “side of membrane” were enriched in low-ROCK1 group and while KEGG pathways including “cytokine receptor interaction”, “hematopoietic cell lineage”, and “primary immunodeficiency” were highly related to low expression of ROCK1 in PMOP patients. The results revealed that low-ROCK1 may interact with proteins and cells next to the plasma, regulating the immune reaction and the expression and function of immune molecules, resulting in the development of PMOP. Bone versus immune system was first reported in 2000 and T cells reaction may influence bone health [65, 66]. Moreover, the immuno-skeletal interface (ISI) has been widely studied for the pathogenesis of osteoporosis, and the role of the immune system in impaired bone turnover was also illustrated especially associated with RANK/RANKL pathways [67]. Therefore, we evaluated the immune infiltration levels in PMOP and found the possible immune cells correlated with ROCK1 through xCell immune infiltration analysis. In PMOP patients, basophils and mast cells decreased whereas DCs and NKT cells increased statistically significantly and positively associated with the low expression of ROCK1. The innate immune, including DCs, has been proven to play an important role in osteoporosis. DCs can impact the skeletal system by producing pro-inflammatory cytokines and transdifferentiating into osteoclasts with the involvement of IL-17, RANKL, or M-CSF [68]. It has been reported that NKT cells can directly produce RANKL and M-CSF, increase the levels of IL-15, and promote osteoclastogenesis [66]. Moreover, invariant NKT (iNKT) cells can activate myeloid dendritic cells and innate immune reactions, further enhancing the development of osteoclasts and boosting bone remodeling in osteoporosis. The role of iNKT was also identified in 79 whole blood samples from PMOP patients, Patients with PMOP exhibit an eight times overexpression of RANKL in their iNKT cells, which may be a significant factor in their bone loss [69]. Consistent with the mRNA results, scRNA-seq illustrated a high expression of ROCK1 in GMP, Monocytes, Neutrophils, and T cells among clustered 10 cell types. However, more research is required to ascertain how ROCK1 influences the pathogenesis of PMOP via a series of immunological responses.

Cancer is a global health challenge, ranking as the second leading cause of mortality and lacking effective treatment strategies. In 2023, the American Cancer Society reported 1,958,310 new cancer cases and 609,820 cancer-related deaths in the United States alone [15]. The incidence of most cancers increases with age, with cancer representing the leading cause of death for individuals aged 60-79 years, who are at an increased risk of developing postmenopausal osteoporosis (PMOP) [15]. Numerous studies have investigated the link between osteoporosis and various types of cancer, particularly breast cancer, where patients with breast cancer, lung cancer, genitourinary cancer, and skin cancer have a higher prevalence of low bone mineral density (BMD) and osteoporosis [17, 70]. Cohort studies with large populations were consistent with the above results [29, 30]. Therefore, we aimed to further investigate the role of ROCK1, the hub gene identified in PMOP, in the context of pan-cancer, with the potential to facilitate early detection and precise therapeutic intervention for various types of cancer.

We found a lower expression of ROCK1 in a total of 18 tumors than in the normal tissues. Along with the clinical studies mentioned above, PMOP-related cancers including breast cancer, lung cancer, and genitourinary cancers especially those in the uterus and ovary showed a low ROCK1 expression. Moreover, the prognosis of SKCM was protected by ROCK1 according to the OS time and K-M curves. A previous study has proven Y-27632, a ROCK1 inhibitor can promote the growth and migration of human melanoma cells *in vivo* [71]. Immunohistochemistry on aggressive/advanced melanoma tissues from 129 patients also showed a down-regulation of Rho kinase signaling with decreased expression of ROCK1 [72]. Thus, low-expression ROCK1 may take part in the development and progression of SKCM in PMOP patients [73]. However, glucocorticoids can promote migration, invasion, and metastasis of melanoma by activating the ROCK1/2, indicating the role of ROCK1 in SKCM was still unclear. We also noticed that ROCK1 in kidney cancers, including KIRP and KICH, was decreased. The expression of ROCK1 in KIRC, which was another common renal tumor, was associated with low OS time and higher levels in T stage, M stage, Stage, and Grade. However, previous studies only focus on renal cell carcinoma (RCC), and multiple miRNAs (such as miR-126, miR-199a, and miR-584) impaired the progression of RCC targeting ROCK1 [74–76]. To confirm ROCK1’s possible function in the treatment of renal cancers, more research on the three renal tumors mentioned in this study should be conducted. We then perform immune infiltration by TIMER and ESTIMATE in pan-cancer and the result showed a negative correlation between DCs and ROCK1 in SARC, which was consistent with xCell immune infiltration analysis in PMOP. circROCK1-E3/E4 was down-regulated in osteosarcoma (OS) and positively associated with the bad prognosis of OS patients [77]. Whether low-ROCK1 expression in PMOP influences the immune microenvironment and then alters the tumor’s features should be further investigated.

There are also some limitations in our study. Firstly, the clinical features of patients with PMOP and pan-cancer were not taken into careful consideration, as the patient data were obtained from public databases. Therefore, further validation of our findings in larger, more diverse cohorts with detailed clinical information is warranted. Secondly, the lack of validation in animal models or patient samples due to the limitations of bioinformatics precludes the assessment of the reproducibility and generalizability of our results. Finally, future research with larger sample sizes is necessary to establish the diagnostic utility of the identified hub genes in PMOP.

## MATERIALS AND METHODS

### Data sources

GSE56815 and GSE7429 were obtained from the Gene Expression Omnibus (GEO, https://www.ncbi.nlm.nih.gov/geo/) database as the training datasets of PMOP. Microarray expression data of controls and PMOP patients in GSE56814 was also acquired from the GEO database to validate the mRNA expression levels and the diagnosis value of genes. The information of microarray datasets was listed in Supplementary Table 1 [78, 79]. GSE147287 was downloaded for single-cell analysis. Pan-cancer data of multiple cancer types, including gene expression levels, survival time, and prognostic data were provided by The Cancer Genome Atlas (TCGA) database (https://portal.gdc.cancer.gov/) and The Therapeutically Applicable Research to Generate Effective Treatments (TARGET, https://ocg.cancer.gov/programs/target/). Normal tissue expression data from the GTEx database (https://gtexportal.org/home/) were used for controls compared with the pan-cancer data.

### Identification of differentially expressed genes

R package “sva” was used to eliminate the batch effect of two PMOP datasets and combine GSE56815 and GSE7429 together as a training dataset for the following analysis [80]. With the criteria of the adjusted p-value (FDR method) <0.05 and |log fold change value| >0.03, we identified the differentially expressed genes (DEGs) between controls and the PMOP patients by “limma” package [81]. Volcano plot was plotted using the “ggplot2” package and the “pheatmap” package was used to visualize the results of DEGs screening with a clustering heat map.

### Weighted gene co-expression network analysis (WGCNA)

WGCNA can be used to identify highly synergistic sets of genes, distinguish genes into modules by analyzing the association relationships between genes, and later look for molecular features of specific phenotypes by correlation analysis between specific modules and sample phenotypes. We used the R package “WGCNA” for the whole analysis and constructed the network [19]. The samples were organized into clusters to detect and remove significant outliers. Co-expression networks were then developed using automatic networks. Then, we identified the soft thresholding power β, which was used to calculate the adjacency of the co-expression network. To select the important key modules as candidates, we performed hierarchical clustering and dynamic tree cut function while gene significance (GS) and module membership (MM) were assessed to link modules with clinical features. Modules having the highest Pearson module membership correlation (MM) and p <0.05 were chosen as the matching modules, and the gene lists of each module were used for further study.

### Functional enrichment analysis

Disease Ontology (DO, https://disease-ontology.org/) is a standardized ontology designed to provide consistent, reusable, and sustainable descriptions of human disease terminology, phenotypic traits, and related medical vocabulary disease concepts. Gene Ontology (GO, http://geneontology.org/) database includes three term groups: biological processes (BP), molecular functions (MF), and cellular components (CC). According to the specific genes, GO enrichment analysis can show the result of several significant GO terms. Kyoto Encyclopedia of Genes and Genomes (KEGG, https://www.kegg.jp/) database aids in comprehending the essential features and purposes of biological systems like cells, organisms, and ecosystems. Using the R package “clusterProfiler”, We perform DO, GO, and KEGG functional enrichment analyses to explore the biological functions, signaling pathways, and diseases that may be highly correlated with DEGs or hub genes [82]. We choose the top significant terms with the adjusted p <0.05 for visualization using bar plots and dot plots by “ggplot2” package.

Gene Set Enrichment Analysis (GSEA, http://www.gsea-msigdb.org/gsea/index.jsp) is a new computational method without appointing the different expression genes, which can include genes with insignificant differential expression but important biological significance that were easily omitted from GO/KEGG enrichment analysis. We calculate the Normalized Enrichment Score (NES) and the p-value to select key terms for the following analysis. R package “clusterProfiler” was used for calculating and showing the results [82].

### Machine learning

With a wide range of applications in the biomedical field, Machine Learning is an important tool belonging to artificial intelligence. We use five types of Machine Learning methods to predict the key hub genes which were greatly associated with PMOP. Support Vector Machines-Recursive Feature Elimination (SVM-RFE) is an algorithm that selects the feature genes with the highest score through multiple iterations. R packages “e1071”, “kernlab” and “caret” were used for SVM-RFE [83, 84]. Using “glmnet” package, we construct the Least Absolute Shrinkage and Selection Operator (LASSO) regression model that selects and regularize the variables to improve the predictability and interpretability of identifying the hub genes [85]. Random Forest (RF) is an algorithm based on ensemble learning, using several decision trees to output the learning result. We use R package “randomForest” to perform the calculation and extrapolate hub genes. Gradient Boosting Machine (GBM) was used to predict the key genes by “gbm” package and genes whose importance scores >0 were considered as the feature genes. eXtreme Gradient Boosting (XGboost) algorithm has high calculating speed and efficiency in screening the phenotype-associated genes. R package “xgboost” was used to set the model and complete the genes prediction. According to the screened genes of each Machines Learning method, we construct a Venn plot by jvenn (http://jvenn.toulouse.inra.fr/app/index.html) to get the common genes as the key hub genes for further study [86]. The correlation between the key hub genes identified was calculated based on the gene expression levels from PMOP datasets with the help of R plot “corrplot”.

### Protein-protein interaction (PPI) network construction

Given a list of query genes, GeneMANIA (https://genemania.org/) can use a large amount of genomics and proteomics data to discover functionally similar genes or proteins and weights each functional genomic dataset according to the predicted values of the query [87]. We use GeneMANIA to explore the co-expression of proteins and construct the PPI networks of hub genes in our study.

### The receiver operating characteristic (ROC) curve analysis and expression analysis

We perform the ROC curve analysis to evaluate the diagnostic value of the hub genes. The ROC curve’s proximity to the upper left corner increases the true positive rate and sensitivity of the test while decreasing the false positive rate and misdiagnosis rates, indicating a better diagnostic value of the genes for PMOP. The ROC curve was assessed and drawn using the "pROC" R package [88]. AUC (Area Under Curve) is the region that the ROC curve encompasses, and axis and hub genes were considered helpful in screening the disease if their AUC was greater than 0.7.

### Immune infiltration analysis

Immune cells play an important role in various diseases and abnormalities. xCell (https://xcell.ucsf.edu/) is a gene signatures-based tool learned from thousands of pure cell types from various sources which can calculate the expression of 64 types of immune and stroma cells in each sample [21]. The level of immune infiltration in controls and PMOP patients was evaluated with the R package “xCell”. We calculated the related coefficient and estimated the correlation between the expression of each immune cell by the “corrplot” package. The correlation between the hub genes and immune cells was also identified by “corrplot”.

### Single-cell analysis

The data of single-cell RNA sequencing (scRNA-seq) on BM-derived mononuclear cells (BM-MNCs) from a PMOP patient was collected from GSE147287. We use R package “Seurat” to conduct quality control, expression data normalization, dimension reduction, and clustering [89]. Cell type annotation was automatically completed by “SingleR” package [90]. R packages “ggplot2” and “cowplot” visualized the analysis result.

### Pan-cancer analysis

After eliminating cancer data with less than three samples, we extracted gene expression data of each sample from the uniformly standardized pan-cancer dataset containing 34 cancer types. log2(x+1) transformation was performed for each expression value and unpaired Wilcoxon Rank Sum and Signed Rank Tests were used for differential analysis. The Human Protein Atlas (HPA) database (http://www.proteinatlas.org/) was used to verify the expression of ROCK1 at the protein level.

A total of 44 cancer species were eventually acquired for investigation after cancer species with fewer than 10 samples in each cancer species were excluded from the data set. We created a Cox proportional hazards regression model using the “coxph” function of the R package “survival” to examine the correlation between gene expression and prognosis in each tumor. The log-rank test was utilized as a statistical test to determine the prognostic significance.

From the pan-cancer dataset, we retrieved the expression data of the target gene and 44 marker genes for RNA modification (m1A, m5C, and m6A) in each sample, and then we did a log2(x+1) transformation. Additionally, modification functions are used to categorize marker genes (writer, reader, eraser). The Pearson correlation between the target gene and marker genes was then calculated using R software (Version 4.2.2).

We took the target gene expression information and the gene expression profile for each tumor from the pan-cancer dataset, mapped the expression profile to Gene Symbol, and then used the R package “ESTIMATE” to compute the immunological ratings for each patient in each tumor based on the gene expression [91]. Taking gene expression into account, we also assessed each patient’s B cell, T cell CD4, T cell CD8, Neutrophil, Macrophage, and DC immune infiltration score in each tumor using the TIMER method of the R package "IOBR" [92, 93]. We calculated the Pearson’s correlation coefficient of the gene and immune infiltration score in each tumor using the “corr.test” function of the R package “psych” to ascertain the significance of the relationship.

We explore the relationship between the gene expression levels and clinical staging of pan-cancer, including T stage, N stage, M stage, Stage, and Grade. The difference in gene expression in each tumor sample from various clinical stages was calculated using R software (version 4.2.2), and the significance of the difference between pairs was analyzed using the unpaired Student’s t-Test and multiple groups of samples using the analysis of variance.

We get the entire Simple Nucleotide Variation data set for level four TCGA samples from GDC (https://portal.gdc.cancer.gov/). The TMB (Tumor Mutation Burden) of each tumor was determined using the “tmb” function of the R package “maftools” [94]. We also gathered the MSI (Microsatellite Instability) score for every tumor from earlier research. We combined the samples’ TMB, MSI, and gene expression data, and for each tumor, we estimated Pearson correlations with the TMB or MSI.

### Data availability statement

The datasets GSE7429, GSE56814, GSE56815, and GSE174287 for this study can be found in the GEO database (https://www.ncbi.nlm.nih.gov/geo/). Pan-cancer data for this study can be found in the TCGA database TARGET database (https://ocg.cancer.gov/programs/target/), and the GTEx database (https://gtexportal.org/home/). Further inquiries can be directed to the corresponding author.

## Supplementary Material

Supplementary Figures

Supplementary Tables
