# Supplementary material for: Identification of ROCK1 as a novel biomarker for postmenopausal osteoporosis and pan-cancer analysis
Source: Aging (Albany NY). 2023 Sep 7;15(17):8873–907. doi: 10.18632/aging.205004 (PMC10522383; doi:10.18632/aging.205004)
Supplement: Supplementary Figures [file aging-15-205004-s001.pdf]

SUPPLEMENTARY FIGURES

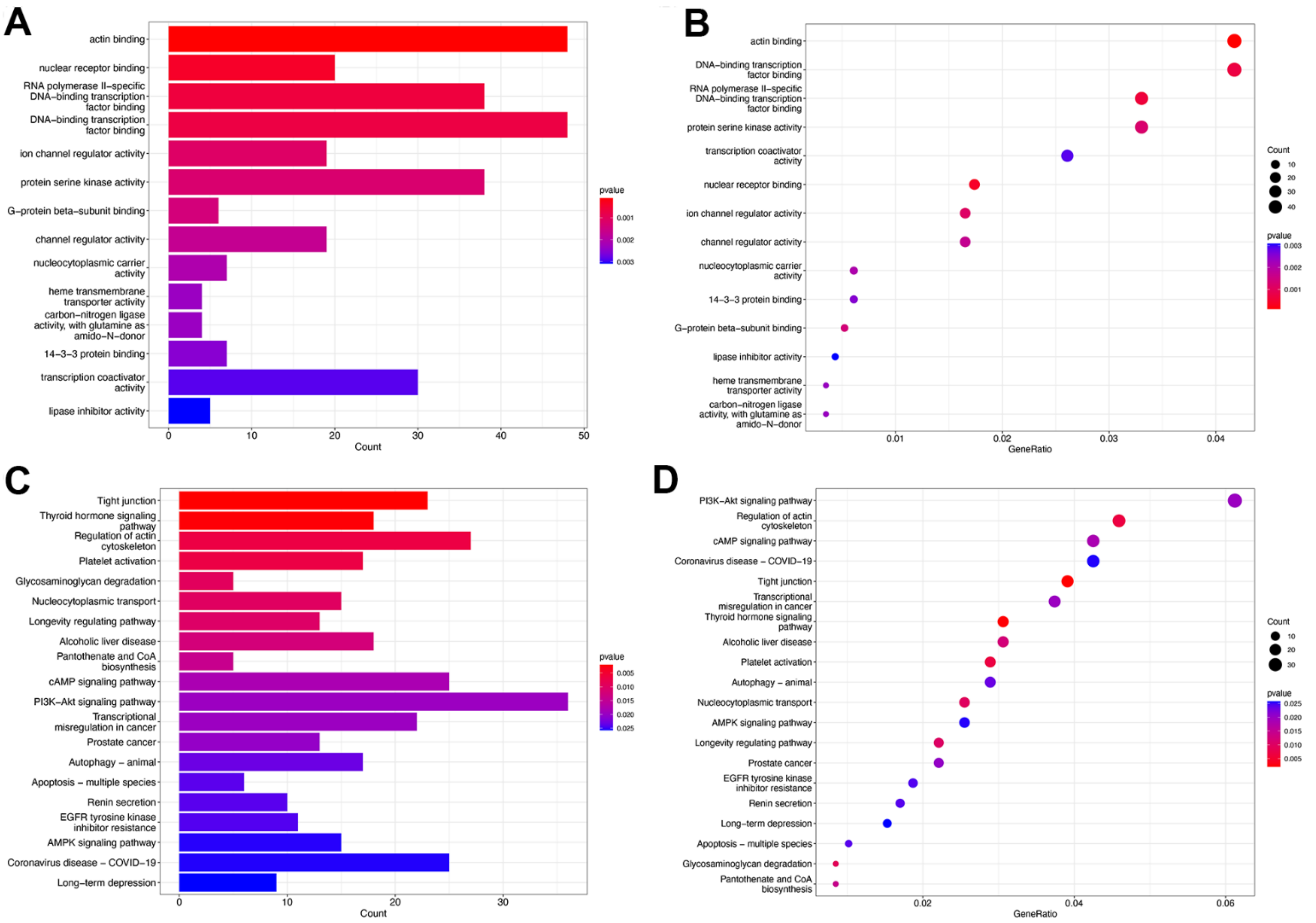

Supplementary Figure 1. Bar plot (**A**) and dot plot (**B**) showed the results of GO enrichment analysis of DEGs. Bar plot (**C**) and dot plot (**D**) showed the results of KEGG enrichment analysis of DEGs.

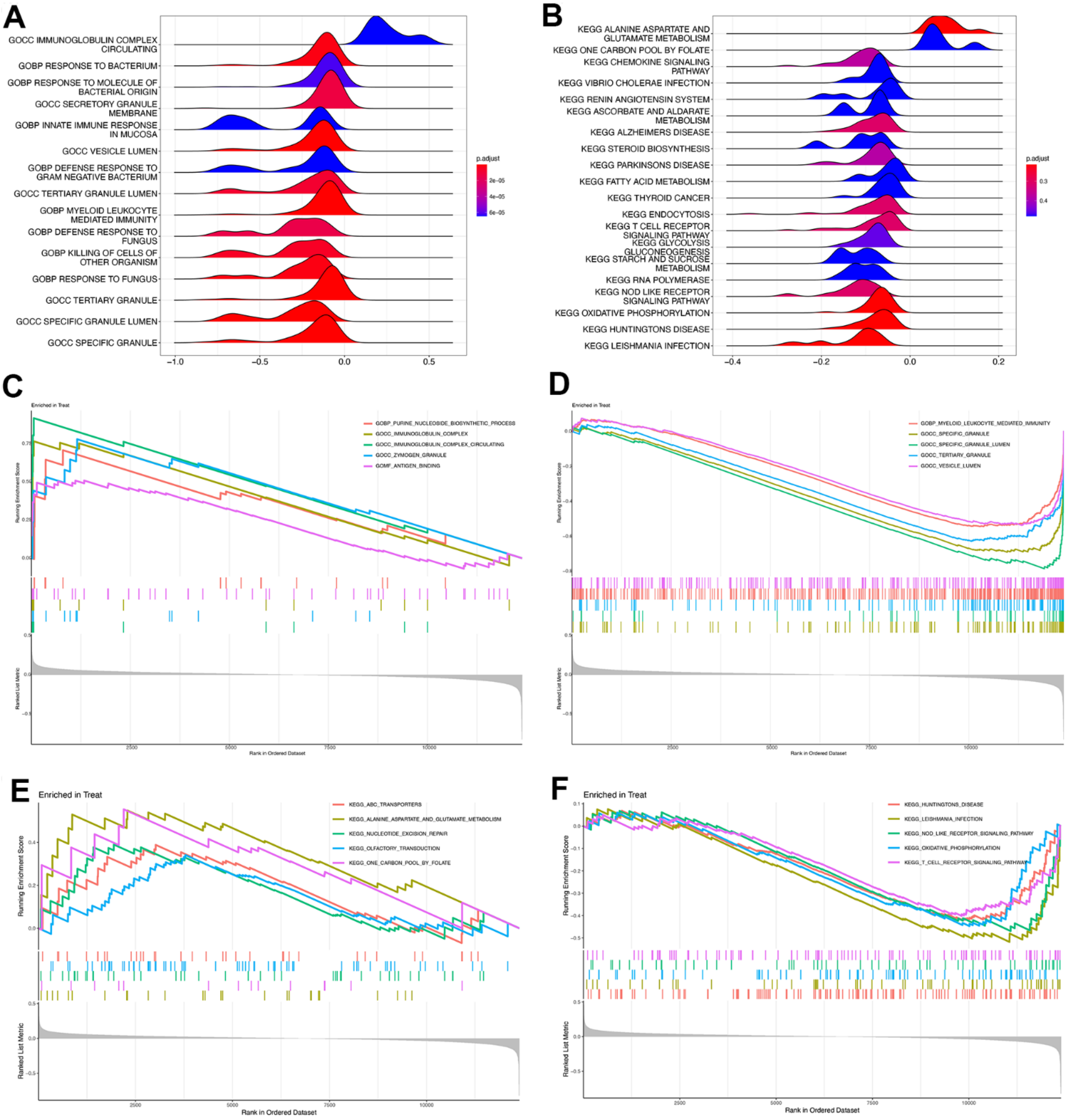

Supplementary Figure 2. Ridge map showed the GO (**A**) and KEGG (**B**) enrichment analysis results by GSEA. GSEA plot with 112 ranked common genes and the top 5 up-regulated GO terms (**C**) or KEGG terms (**D**). GSEA plot with 112 ranked common genes and the top 5 down-regulated GO terms (**E**) or KEGG terms (**F**).

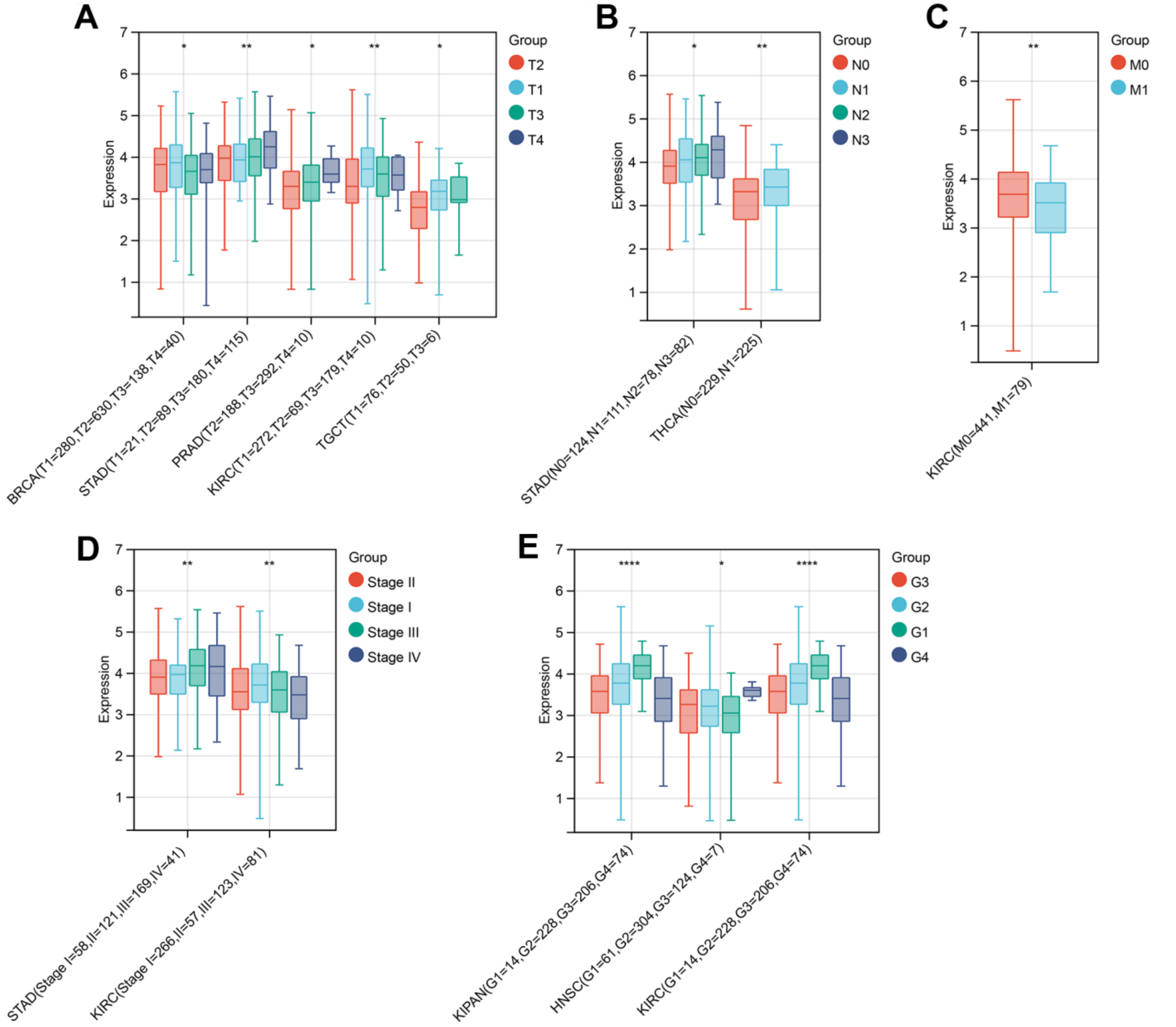

Supplementary Figure 3. Significantly different Pan-cancer expression levels of ROCK1 in T-stage (**A**), N-stage (**B**), M-stage (**C**), Stage (**D**), and Grade (**E**). *p <0.05, **p <0.01, ***p <0.001, ****p <0.0001.

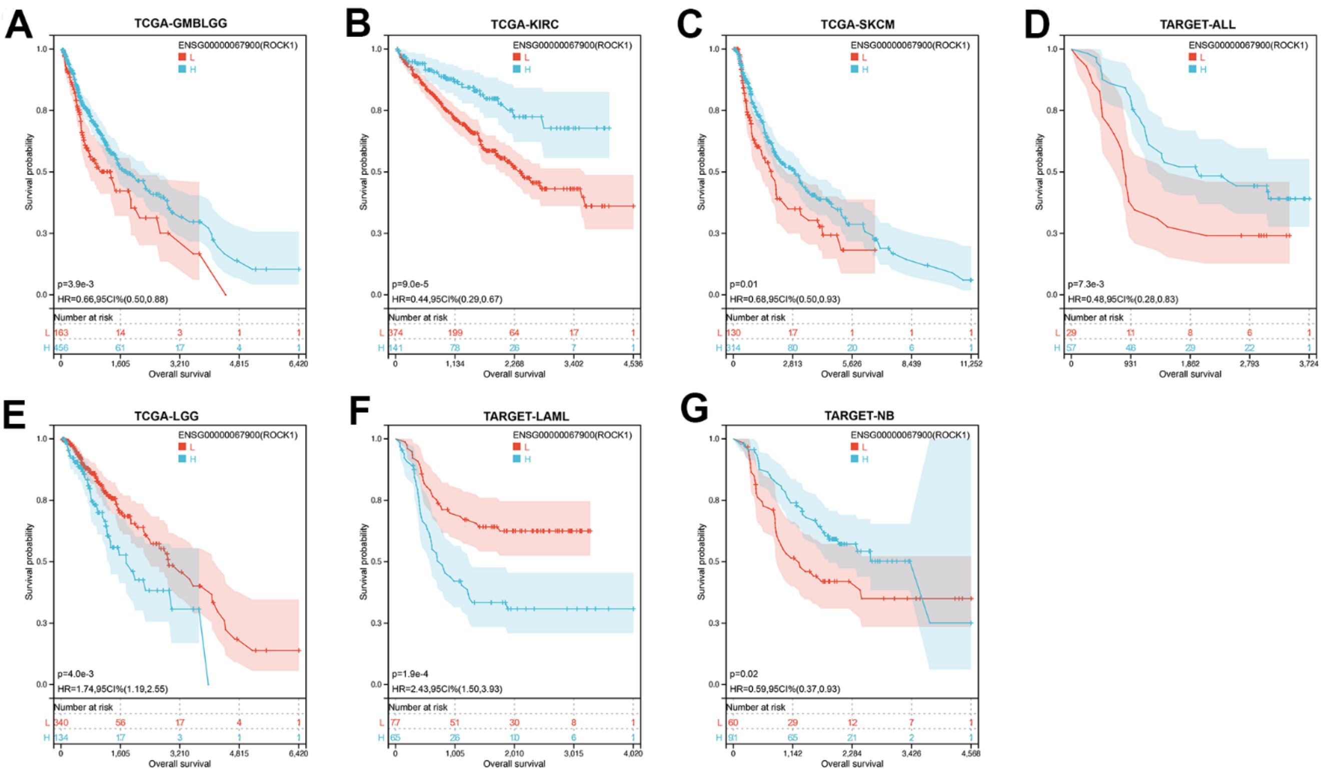

Supplementary Figure 4. Analysis of the overall survival in multiple tumors with the best cut-off method. The expression levels of ROCK1 are positively correlated with OS in TCGA-GMBLGG (**A**), TCGA-KIRC (**B**), TCGA-SKCM (**C**), and TARGET-ALL (**D**). The expression levels of ROCK1 are negatively correlated with OS in TCGA-LGG (**E**), TCGA-LAML (**F**), and TARGET-NB (**G**).

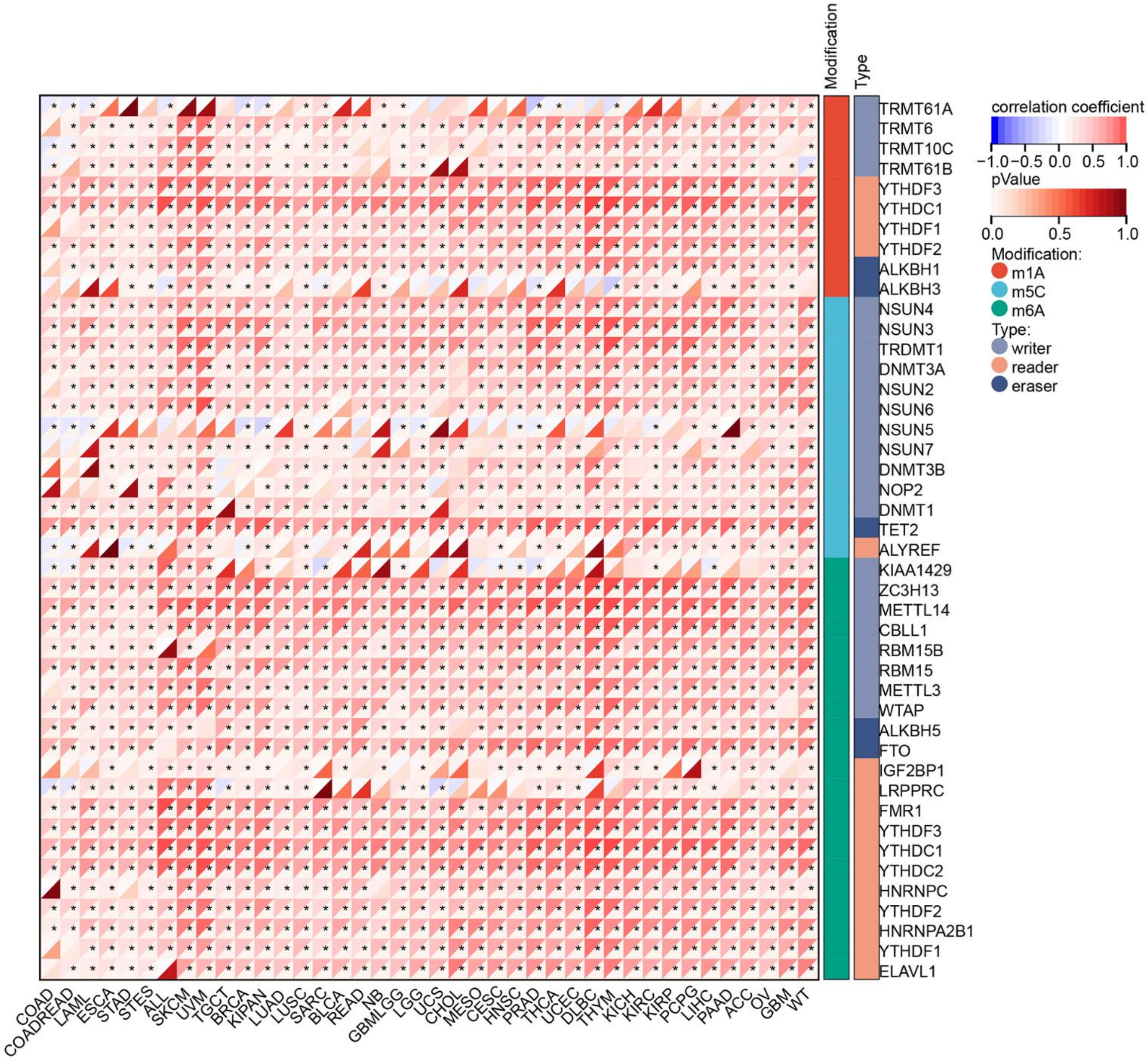

Supplementary Figure 5. Correlation of ROCK1 expression with RNA-modified (m1A-, m5C-, and m6A-modified) gene expression in multiple cancers. *$p < 0.05$

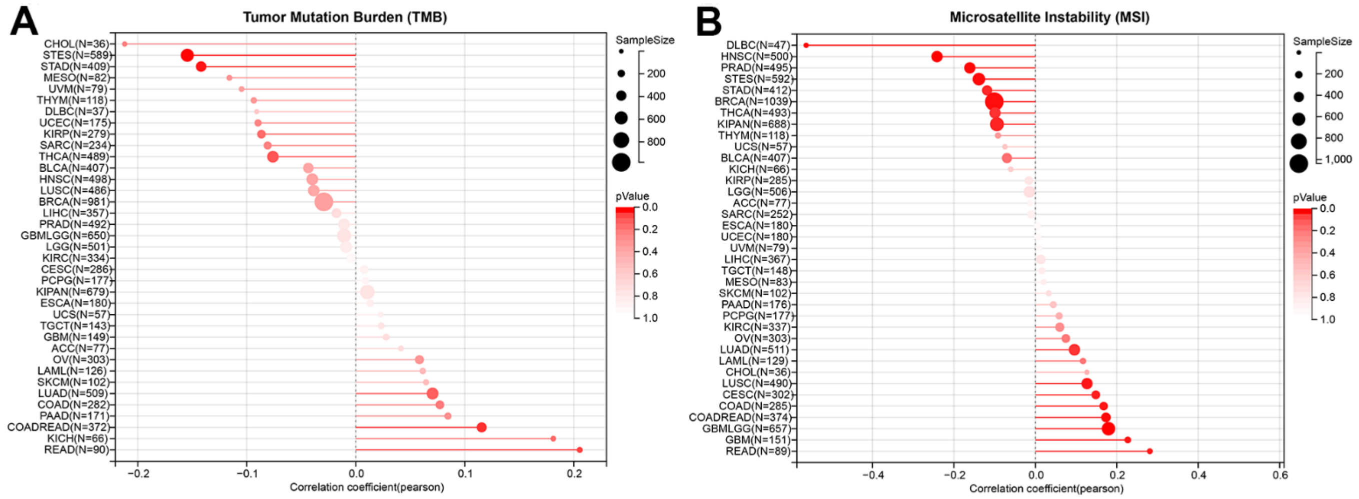

Supplementary Figure 6. (**A**) Correlation between the expression levels of ROCK1 and TMB pan-cancer. (**B**) Correlation between the expression levels of ROCK1 and MSI pan-cancer.
