# Supplementary material for: Identification of ROCK1 as a novel biomarker for postmenopausal osteoporosis and pan-cancer analysis
Source: Aging (Albany NY). 2023 Sep 7;15(17):8873–907. doi: 10.18632/aging.205004 (PMC10522383; doi:10.18632/aging.205004)
Supplement: Supplementary Tables [file aging-15-205004-s002.pdf]

SUPPLEMENTARY TABLES

Supplementary Table 1. The characteristics of microarray datasets obtained from GEO database.

GEO Dataset	Platform	PMOP(n)	Control(n)	Year	Country	PMID
GSE56815	GPL96	40	40	2016	USA	29330445
GSE7429	GPL96	10	10	2008	USA	18433299
GSE56814	GPL96	31	42	2016	USA	30056508, 31073748, 29330445

Supplementary Table 2. Basic information about patients from whom samples for IHC staining in the HPA database.

Position	Tissue	Patient ID	Sex	Age	Staining
Skin	Normal	1876	Female	46	High
	Cancer	2013	Female	85	Low
Kidney	Normal	3521	Female	68	Medium
	Cancer	1498	Female	70	Not detected
Lung	Normal	2268	Female	49	High
	Cancer	3016	Female	73	Not detected
Breast	Normal	3544	Female	45	Medium
	Cancer	2083	Female	51	Low
Cervix	Normal	2102	Female	57	Medium
	Cancer	4218	Female	29	Not detected
Ovary	Normal	2264	Female	60	Medium
	Cancer	2082	Female	57	Low
